# Stable simulations do not guarantee functional engagement: a case study of off-target prediction for Seladelpar and Zanamivir

**DOI:** 10.1007/s10822-026-00936-w

**Published:** 2026-09-03

**Authors:** Rachel Kemp, Thomas J. Kean, Kirill E. Medvedev

**Affiliations:** 1 Biionix (Bionic Materials, Implants and Interfaces) Cluster, Department of Medicine, University of Central Florida College of Medicine, Orlando, FL 32826 USA; 2 Burnett School of Biomedical Sciences, University of Central Florida, Orlando, FL 32827 USA; 3 Department of Computer Science, University of Central Florida, Orlando, FL 32816 USA; 4 Genomics & Bioinformatics Cluster, University of Central Florida, Orlando, FL 32816 USA

**Keywords:** Off-target prediction, Protein domains, AlphaFold3, Molecular dynamics, Drug repurposing

## Abstract

Identifying off-target interactions of approved drugs is important to anticipate side effects and uncover repurposing opportunities. Computational pipelines combining structural homology, structure prediction, and molecular dynamics (MD) simulations offer a promising strategy, but it remains unclear whether stable, control-like MD trajectories reliably indicate functional engagement. We examined this in a case study of two approved drugs. Using the Evolutionary Classification of Protein Domains (ECOD) framework to select candidate off-targets, we modeled each drug-protein complex as two independent AlphaFold3 models and simulated both by MD, for Seladelpar (a PPARδ agonist) and Zanamivir, an influenza neuraminidase inhibitor that also inhibits human Sialidase-2 (NEU2). Candidates were ranked by the similarity of global MD descriptors to the on-target control. For Seladelpar, the three top-ranked candidates (FXR, RARγ, ERRγ) were tested experimentally; the Zanamivir set was analyzed computationally only. None showed measurable activity in reporter or thermal shift assays, despite stable simulations and descriptor values comparable to the control. Including PPARα and PPARγ as weak-positive comparators, these descriptors did not rank genuine interactions closer to the control than inactive candidates. Residue-level comparison with experimental structures showed the predicted poses reproduced only part of the canonical contacts. Where experimental drug-bound structures existed, AlphaFold3 reproduced the pose for PPARα but not PPARγ, and its per-model confidence did not track pose accuracy. Within this case study, the specific global descriptors examined reflect complex stability rather than functional engagement, which does not mean MD-based approaches cannot make this distinction.

**Supplementary Information:**

The online version contains supplementary material available at https://doi.org/10.1007/s10822-026-00936-w.

## Introduction

Small-molecule drugs rarely act exclusively on a single protein. Beyond their direct target, most small-molecule drugs also interact with other proteins, and these off-target interactions can produce important biological consequences including adverse side effects [1]. Identifying a drug’s off-target interactions is valuable not only for anticipating side effects, but also for uncovering potential repurposing opportunities. As a result, computational methods have been explored for the prediction of off-target interactions prior to experimental testing [2]. Several computational strategies are routinely applied to this problem, including ligand-based similarity searches, molecular docking, and structure-based binding-site comparison. Although these approaches are fast and have produced meaningful predictions, methods based on a single static structure or on overall similarity have known limitations [3, 4]. Molecular dynamics (MD) simulation addresses some of these limitations through temporal modeling of a drug-protein complex. Rather than relying on a single structural snapshot, MD captures the stability and dynamic behavior of a predicted binding mode, and trajectory-derived measures have been used to assess whether a predicted complex is likely to represent a biologically relevant interaction.

Another requirement for off-target prediction is a systematic method to select candidate proteins for experimental evaluation. Proteins with structurally similar or evolutionarily related domains can bind chemically similar ligands, which makes structural domain classification a rational basis for selecting candidate off-targets [5]. The Evolutionary Classification of Protein Domains (ECOD) provides exactly this kind of organizing framework. ECOD is a hierarchical classification of protein domains of known structure that groups domains by evolutionary relationship rather than by fold similarity alone, thereby prioritizing homology over topology [6]. It is also the most comprehensive protein domain classification system currently available, encompassing not only the largest set of experimental structures from the Protein Data Bank (PDB) [7] but also millions of domains derived from AlphaFold-predicted models [6, 8, 9]. Within the ECOD hierarchy, the topology (T-group) level subdivides homologous domains into groups that also share the same topology. T-groups thus provide well-defined sets of closely related proteins likely to share ligand-binding properties. Building on this framework, we previously developed DrugDomain, the first database linking small-molecule ligand binding to evolutionary domain classification [10, 11]. Organizing ligand-binding data by domain classification enables the identification of structural homologs of a drug’s target as candidate off-targets – the approach we model, simulate, and experimentally test in the present work.

In this study, we applied a pipeline integrating ECOD-based candidate selection, AlphaFold3 modeling [12], and molecular dynamics (MD) simulation to two approved drugs: Seladelpar, an agonist of peroxisome proliferator-activated receptor delta (PPARδ), and Zanamivir, an influenza neuraminidase inhibitor that also inhibits human Sialidase-2 (NEU2). For each drug, off-target candidates were selected from members of the ECOD T-group containing its primary target: the nuclear receptor ligand-binding domain and the 6-bladed β-propeller ECOD T-groups, respectively. As positive comparators for the Seladelpar system, we additionally included PPARα and PPARγ, the two remaining PPAR subtypes, which are weakly activated by Seladelpar. Each drug-protein complex was modeled and simulated as two independent AlphaFold3 models, covering eleven complexes for the nuclear receptor T-group and seven for the 6-bladed β-propeller T-group. Clustering of MD trajectory features was used to rank the candidates by their similarity to the on-target control, and the top-ranked predictions for Seladelpar were tested experimentally using reporter activity assays and thermal shift binding assays. None of the three top-ranked nuclear receptor candidates (FXR - Farnesoid X receptor, RARγ - Retinoic acid receptor gamma, and ERRγ - Estrogen-related receptor gamma) showed measurable functional activity, despite stable simulations and trajectory metrics comparable to the on-target PPARδ control. Structural comparison of the AlphaFold3-predicted binding modes with experimental agonist-bound structures provides a plausible explanation for this discordance. In FXR and RARγ, AlphaFold3 placed Seladelpar’s carboxylate at the carboxylate-recognition site used by that receptor’s own agonist, but reproduced only part of the canonical activating contacts. Where an experimental Seladelpar structure was available it reproduced the binding pose for PPARα but not PPARγ. These results show that the global trajectory descriptors used here capture the stability of a predicted complex but not whether its binding mode supports receptor activation, and that distinguishing off-targets from false positives may require residue-level structural comparison with experimental agonist-bound structures.

## Materials and methods

### Data preparation and collection

For the current study, we aimed to select two FDA-approved drugs, each targeting proteins from a different ECOD (v292, 08302024) homology group (H-group) [6, 8]. We excluded membrane proteins because simulating them requires embedding in an explicit lipid bilayer, which substantially increases system size, requires longer equilibration, and would have made these systems non-comparable to the soluble complexes simulated under a single uniform protocol here. We also excluded protein kinases, whose polypharmacology is already characterized in depth: the family is exhaustively catalogued structurally [13], and inhibitor specificity has been a central focus of the field for two decades [14, 15]. We considered only human protein targets with determined experimental 3D structure.

The first selected pair of drug-target is Peroxisome proliferator-activated receptor (PPAR) delta with Seladelpar (DrugBank accession: DB12390; PDB ligand id: KKB), whose ligand-binding domain belongs to ECOD T-group 188.1.1 (Nuclear receptor ligand-binding domain). The starting point for the first system was the experimental structure of PPARδ with Seladelpar (8huo), whose ECOD domain (e8huoA1) defines the T-group searched for candidates. Candidates were selected from the ECOD representative domains of this T-group, which contains two F-groups with experimental structures: 188.1.1.1 and 188.1.1.2. From 188.1.1.1 we selected seven of the available representative domains, and from 188.1.1.2 the single available representative domain, giving eight candidates (Table 1). Sequence identity to the PPARδ ligand-binding domain ranged from 24.5 to 29.2% across the eight candidates, compared with 70.5% for PPARα and 62.6% for PPARγ. Identity was not used as a selection criterion, since ECOD F-group and T-group membership already establishes homology. The experimental structures of Seladelpar bound to PPARα (8hun) and PPARγ (8hup) are not among the ECOD representative domains and were therefore not part of the initial candidate set. Both were subsequently added as comparators with known Seladelpar activity (Sect.  3.1).

**Table 1:** List of potential targets for Seladelpar from Nuclear receptor ligand-binding domain ECOD T-group

PDB	UniProt	Organism	Name	ECOD Fid	ECOD domain	Seq. identity to PPARδ (%)
8huo (control)	Q03181	Human	Peroxisome proliferator-activated receptor delta (PPARδ)	188.1.1.1	e8huoA1	100
1fcy	P13631	Human	Retinoic acid receptor gamma (RARγ)	188.1.1.1	e1fcyA1	27.3
1ie9	P11473	Human	Vitamin D3 receptor (VDR)	188.1.1.1	e1ie9A1	28.7
1osh	Q96RI1	Human	Bile acid receptor (FXR)	188.1.1.1	e1oshA1	28.3
1pq9	P55055	Human	Oxysterols receptor LXR-beta (LXRβ)	188.1.1.1	e1pq9A1	29.2
1xvp	P19793	Human	Retinoic acid receptor RXR-alpha (RXRα)	188.1.1.1	e1xvpB1	27.8
2e2r	P62508	Human	Estrogen-related receptor gamma (ERRγ)	188.1.1.1	e2e2rA1	24.5
3hvl	O75469	Human	Nuclear receptor subfamily 1 group I member 2 (PXR)	188.1.1.2	e3hvlA1	27.8
1pzl	P41235	Human	Hepatocyte nuclear factor 4-alpha (HNF4α)	188.1.1.1	e1pzlA1	27.6
8hup	P37231	Human	Peroxisome proliferator-activated receptor gamma (PPARγ)	188.1.1.1	e8hupA1	62.6
8hun	Q07869	Human	Peroxisome proliferator-activated receptor alpha (PPARα)	188.1.1.1	e8hunA1	70.5

The second drug selected was Zanamivir (DrugBank accession: DB00558; PDB ligand id: ZMR), an influenza neuraminidase inhibitor that also inhibits human Sialidase-2 (NEU2) in the micromolar range. The experimental structure of NEU2 with Zanamivir (2f0z), whose ECOD domain e2f0zA1 belongs to T-group 5.1.3 (6-bladed beta-propeller), was used as the reference human complex defining the T-group searched. This T-group is considerably larger and contains many F-groups. We selected human representative domains from its five largest F-groups, giving six candidates (Table 2) F-group 5.1.3.7 contributed two domains. For this system, sequence identity to the NEU2 domain ranged from 21.9 to 26.5% across the six candidates (Table 2), which belong to five different F-groups within T-group 5.1.3. Each candidate required two independent structural models and two MD simulations, so the candidate sets were kept to a tractable size and do not cover every member of the two T-groups. Other members of these T-groups may bind the drugs but were not tested here.

**Table 2:** List of potential targets for Zanamivir from 6-bladed beta-propeller ECOD T-group

PDB	UniProt	Organism	Name	ECOD Fid	ECOD domain	Seq. identity to NEU2 (%)
2f0z (control)	Q9Y3R4	Human	Sialidase-2 (NEU2)	5.1.3.26	e2f0zA1	100
1ijq	P01130	Human	Low-density lipoprotein receptor (LDLR)	5.1.3.2	e1ijqA1	23.7
1zgk	Q14145	Human	Kelch-like ECH-associated protein 1 (KEAP1)	5.1.3.6	e1zgkA1	26.5
4gnb	Q15493	Human	Regucalcin (RGN, Gluconolactonase)	5.1.3.23	e4gnbA1	22.8
4wxq	Q99972	Human	Myocilin (MYOC)	5.1.3.9	e4wxqA1	25.4
7qrw	O75382	Human	Tripartite motif-containing protein 3 (TRIM3)	5.1.3.7	e7qrwA1	23.5
7qrv	Q9C040	Human	Tripartite motif-containing protein 2 (TRIM2)	5.1.3.7	e7qrvA1	21.9

### AlphaFold3 modelling and molecular dynamics simulation protocol

Structural models of each protein-drug complex were generated with AlphaFold3 (v3.0.1) [12], run locally in a container with the default parameter set. Multiple sequence alignments were built against the standard AlphaFold3 databases (BFD, MGnify 2022_05, UniRef90 2022_05 and UniProt 2021_04), and template search used the PDB snapshot of 28 September 2022 with the default maximum template date of 30 September 2021. The experimental Seladelpar complexes 8HUN and 8HUP (deposited December 2022) were therefore absent from both the template database and the AlphaFold3 training set. For each complex, the input consisted of the protein domain sequence, taken from UniProt KB [16] over the residue range of the corresponding ECOD domain, together with the drug specified by its PDB chemical component identifier (KKB for Seladelpar, ZMR for Zanamivir). Each job used a single random seed and produced five predicted samples. No command-line options were changed from their defaults. Template search retained the default maximum template date of 30 September 2021, so structures released after that date were not available to the model. Models were ranked by the AlphaFold3 ranking score, and the two highest-ranked models of each complex were used as independent starting structures for molecular dynamics. Models were also generated for the two reference complexes (PPARδ-Seladelpar and NEU2-Zanamivir), because the corresponding experimental structures contain unresolved regions.

Molecular dynamics simulations were conducted using GROMACS (2025.2) [17]. Protein and ligand topologies were prepared separately. The PDB file containing the structural model of the protein and drug were separated into two files – one containing only the protein and the other containing only the ligand. We used the CHARMM36m force field to prepare protein topology and MD simulations, since it is widely used for its balanced treatment of secondary structure and dynamic properties [18]. GROMACS command *pdb2gmx* was used to generate protein topology with default water model (CHARMM-modified TIP3P [19]). Generation of ligand topology was conducted using CHARMM General Force Field server (CGenFF) [20–22]. Using PyMOL [23], the ligand pdb file was converted to a mol2 file with added hydrogens, which was used as input for the CGenFF server. The CGenFF output .str file was converted to GROMACS format using the CGenFF conversion script [24], after which the ligand parameters were included into the system’s topology (file topol.top).

Each complex was placed in a cubic box with a minimum edge distance of 1.0 nm and solvated. Sodium and chloride ions were added to neutralize the net system charge. Energy minimization used steepest descent with a convergence criterion of 1000 kJ mol⁻¹ nm⁻¹ (maximum 50 000 steps). The systems were then equilibrated for 100 ps in the NVT ensemble at 300 K, followed by 100 ps in the NPT ensemble at 1.0 bar, with position restraints applied to both protein and ligand heavy atoms throughout equilibration. Temperature was maintained with the velocity-rescaling thermostat [25] (τ_T = 0.1 ps, protein-ligand and solvent-ion groups coupled separately) and pressure with the C-rescale barostat (isotropic, τ_P = 2.0 ps, compressibility 4.5 × 10⁻⁵ bar⁻¹) during NPT equilibration. Production simulations were run in the NPT ensemble with the Parrinello-Rahman barostat [26] under the same coupling parameters, using a 2 fs timestep and no position restraints. Bonds involving hydrogen were constrained with LINCS [27] (order 4, one iteration). Van der Waals interactions used a force-switch modifier between 1.0 and 1.2 nm; electrostatics were treated with Particle Mesh Ewald (PME) with a 1.2 nm real-space cutoff, fourth-order interpolation and 0.16 nm grid spacing. Coordinates were saved every 10 ps. Each protein-drug complex was simulated for 30 ns from each of two independent AlphaFold3 models. The PPARα and PPARγ complexes were additionally extended to 100 ns.

### Experimental validation

#### Reporter activity assays

Experimental validation focused on the PPARδ/Seladelpar system, given the availability of well-validated commercial reporter assays for the predicted nuclear receptor off targets including Farnesoid X receptor (FXR), Retinoic acid receptor gamma (RARγ), and Estrogen-related receptor gamma (ERRγ). Human FXR, RARγ, and ERRγ reporter assay kits were obtained from INDIGO Biosciences (State College, PA, USA). All assays were conducted according to the manufacturer’s instructions. For agonist-mode testing, Seladelpar was evaluated across eight concentrations using a 4-fold serial dilution scheme ranging from 1024 to 0.0625 nM, based on the reported EC_50_ value (2 nM [28]; 20.2 nM in the transactivation assay of [29]) for PPARδ activation. The relative light units (RLUs) were recorded 24 h-post drug application using the SpectraMax iD5 Multi-Mode Microplate Reader (Molecular Devices). Activity was assessed in comparison to the provided reference agonists. For antagonist-mode testing in the FXR and RARγ reporter assays, cells were first stimulated with the provided reference agonist, GW4064 at the reported EC_80_ concentration and *trans*-retinoic acid at the reported EC_50_ concentration, respectively, prior to treatment with Seladelpar using the same eight-point dilution scheme described for agonist-mode testing. Reporter cells included in the ERRγ reporter assay constitutively express active ERRγ in the absence of ligand binding and therefore did not require pre-stimulation with a reference agonist. Seladelpar was added directly to the reporter cells using the same dilution scheme described for agonist-mode testing. All assay conditions were performed in triplicate alongside media-only controls.

#### Protein thermal shift assays

Protein thermal shift assays were performed using the Protein Thermal Shift Dye Kit (Thermo Fisher Scientific) according to the manufacturer’s instructions. Protein melt reactions were prepared in an optical 96-well reaction plate using human FXR ligand binding domain GST-tag recombinant protein (Thermo Fisher Scientific) and human RARγ ligand binding domain GST-tag recombinant protein (Thermo Fisher Scientific). Recombinant proteins were added to each well at a concentration of 1 µg per well in the supplied assay buffer containing 1x dye. For ligand-binding conditions, Seladelpar was added to a final concentration of 512 nM per well, corresponding to the highest concentration evaluated in the reporter assays. Negative controls included no-protein controls containing only assay buffer and dye to assess potential reagent contamination, as well as ligand-only controls containing buffer, dye, and Seladelpar to assess potential ligand-dye interactions. All reactions were prepared on ice in triplicate, sealed, and briefly centrifuged. Melt curves were performed using a QuantStudio7 Flex Real-Time PCR system equipped with the QuantStudio Real-Time PCR Software (v1.x). De-coupled optical filter settings were used with excitation at 470 ± 15 nm and emission at 586 ± 10 nm.

### Statistical analysis

To comprehensively characterize protein-ligand binding dynamics, we extracted five key metrics computed with GROMACS tools on trajectories centered on the protein-ligand complex: (1) protein backbone RMSD (Root Mean Square Deviation) with gmx rms (least-squares fit to the starting backbone), (2) protein-ligand center-of-mass (COM) distance with gmx distance, (3) hydrogen bond count with gmx hbond, (4) protein-ligand contact count with gmx mindist (atoms within 0.4 nm), and (5) ligand solvent-accessible surface area (SASA) with gmx sasa. Features were computed over the first 30 ns of each trajectory for all complexes, and additionally over the full 100 ns for the PPARα and PPARγ complexes. For each metric, ten statistical features were computed from the time-series data: mean, standard deviation, median, minimum, maximum, 25th percentile (Q25), 75th percentile (Q75), interquartile range (IQR = Q75 - Q25), coefficient of variation (CV = σ/µ), and range (max - min). This approach yielded a 50-dimensional feature vector per trajectory (5 metrics × 10 statistical features). All features were normalized to the same scale before analysis to prevent metrics with larger values from dominating the results. Principal Component Analysis (PCA) and t-Distributed Stochastic Neighbor Embedding (t-SNE) were applied to the standardized feature matrix. The t-SNE algorithm was implemented with perplexity parameter set to 5, within the recommended range for small datasets and below the theoretical maximum of n−1. The algorithm was run for 1000 iterations with a fixed random seed to ensure reproducibility.

Distances to the control were computed as Euclidean distances in the standardized 50-feature space, taking the nearer of the two control replicates. Replicate consistency was assessed by comparing, for each receptor, the distance between its two independent models with its distance to the nearest control replicate. Per-feature comparisons between the control and the experimentally inactive complexes report the difference in group means scaled by the pooled standard deviation, together with whether the two value ranges overlap. Pairwise feature correlations were computed on the same standardized matrix.

Predicted ligand poses were compared with experimental structures in PyMOL [23]. Each model was superposed on the experimental structure by sequence-based alignment of the protein without outlier rejection (align, cycles = 0). Ligand RMSD was then computed in this frame without further fitting, using the atom correspondence from an alignment of the two ligands performed without transformation. Binding-site Cα RMSD was computed over the experimental binding-site residues, defined as residues with any atom within 5 Å of the ligand in the experimental structure, using the residue correspondence from the protein alignment.

Reporter Activity Assay data was graphed using GraphPad Prism. Fluorescence data from the Protein Thermal Shift Assays was exported and analyzed in GraphPad prism. Derivative curves were plotted to assess the proteins’ melting temperatures, which were determined by nonlinear regression using a Boltzmann sigmoidal model.

## Results and discussion

### ECOD-guided selection of candidate off-targets

**Fig. 1:**
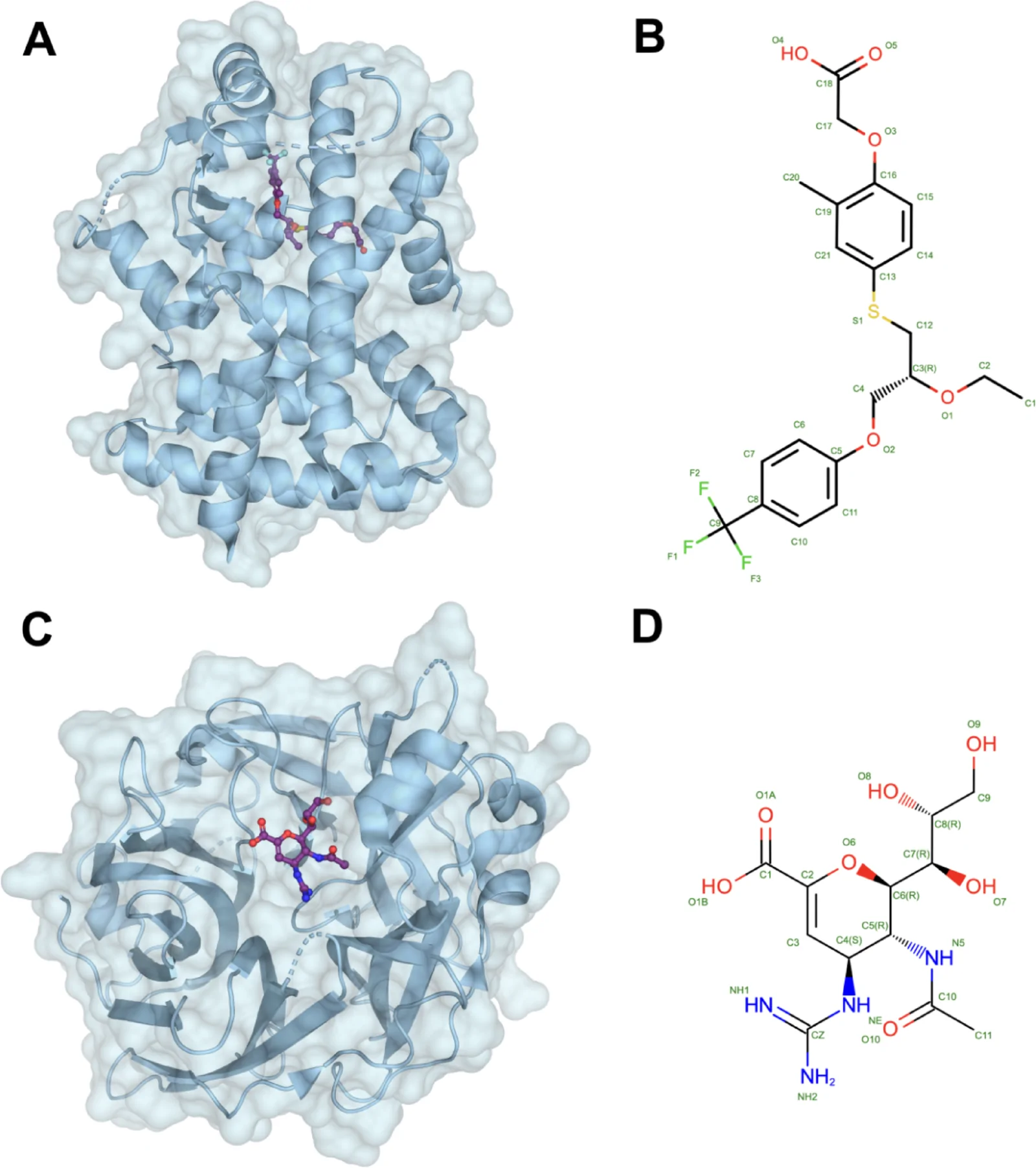
Representative protein examples of ECOD T-groups selected for potential drug-target assessment. **A** Structure of Peroxisome proliferator-activated receptor (PPAR) delta with Seladelpar (PDB: 8huo; ECOD T-group name: Nuclear receptor ligand-binding domain). **B** 2D structure of Seladelpar. **C** Structure of Sialidase 2 with Zanamivir (PDB: 2f0z; ECOD T-group name: 6-bladed beta-propeller). **D** 2D structure of Zanamivir

We selected two ECOD T-groups for verification of potential drug-target interaction (see Materials and Methods). The first ECOD T-group (Nuclear receptor ligand-binding domain) is defined by the experimental structure of Peroxisome proliferator-activated receptor (PPAR) delta with Seladelpar [29] (Fig. 1A, B). PPARs comprise a subfamily of ligand-activated transcription factors within the nuclear receptor superfamily. Seladelpar is an FDA approved PPARδ agonist, used to treat an autoimmune disease of the liver [30]. It reduces bile acid production through downregulation of CYP7A1, the rate-limiting enzyme in bile acid synthesis [31]. PPARδ adopts an α-array architecture, consisting of eleven major α-helices and four β-strands (Fig. 1A). Eight closely related PPARδ homologs representing different receptor types were selected from the same ECOD T-group to assess potential interactions with Seladelpar (Table 1). We additionally included PPARα and PPARγ, the two remaining PPAR subtypes, as comparators with known Seladelpar activity. In transactivation assays, Seladelpar activates PPARδ with an EC50 of 20.2 nM (99.3% efficacy) but PPARα and PPARγ only at micromolar concentrations (1.64 µM, 41.0%; and 3.53 µM, 58.5%, respectively) [29]. These two receptors therefore represent weak but genuine interactions, against which the trajectory-derived rankings can be calibrated. Experimental structures of Seladelpar bound to PPARα (8HUN) and PPARγ (8HUP) are available from the same study [29], allowing the predicted binding modes to be compared directly with the drug-bound crystal structures.

The second ECOD T-group (6-bladed beta-propeller) is represented by the experimental structure of human Sialidase 2 with Zanamivir [32] (Fig. 1C, D). Zanamivir is a neuraminidase inhibitor used to treat influenza A [33] and B viruses [34]. Neuraminidase facilitates the release of newly formed virions, supporting this enzyme a valid drug target. However, it was shown that Zanamivir also inhibits human sialidases NEU2 and NEU3 in the micromolar range, with Ki values of approximately 12.9 µM and 3.7 µM respectively [32]. NEU2 is therefore a validated human off-target of Zanamivir rather than its therapeutic target, and it is used here as the reference human complex for the T-group search because it is the human sialidase for which an experimental Zanamivir structure is available. Therefore, other human proteins closely related to Sialidase 2 could also be affected by Zanamivir binding. Human Sialidase 2 adopts a 6-bladed β-propeller fold comprising six four-stranded β-sheets arranged in a propeller-like architecture. One alpha helix participates in the coordinating of the drug binding (Fig. 1C). Six closely related Sialidase 2 homologs representing different receptors and enzymes were selected from the same ECOD T-group to assess potential interactions with Zanamivir (Table 2).

For the generation of a protein model with its corresponding drug, we used AlphaFold3 [12]. MD simulation production was conducted for 30 ns per trajectory. To verify that this window was sufficient to characterize the complexes, the four PPARα and PPARγ systems were additionally extended to 100 ns. Trajectory-derived features computed over the first 30 ns closely matched those from the full 100 ns trajectory: center-of-mass distance, ligand SASA, and contact counts changed by less than 2%, and the mean backbone RMSD stayed between 1.2 and 2.0 Å, reflecting slow equilibration rather than any late conformational transition (Supplementary Fig. 1). Hydrogen-bond counts fluctuated more (means shifting by up to ~ 35%) but showed no systematic drift, consistent with the sensitivity of this geometric metric to small changes in a low count. This supports the use of a 30 ns window for the current set of complexes. Consistent with this, earlier work has reported that short simulations of 10-50 ns distinguish active from decoy compounds as well as longer runs [35], and that 20 ns is sufficient to assess structural stability and conformational dynamics in both apo and holo forms [36].

### Clustering of complexes by trajectory-derived features

**Fig. 2:**
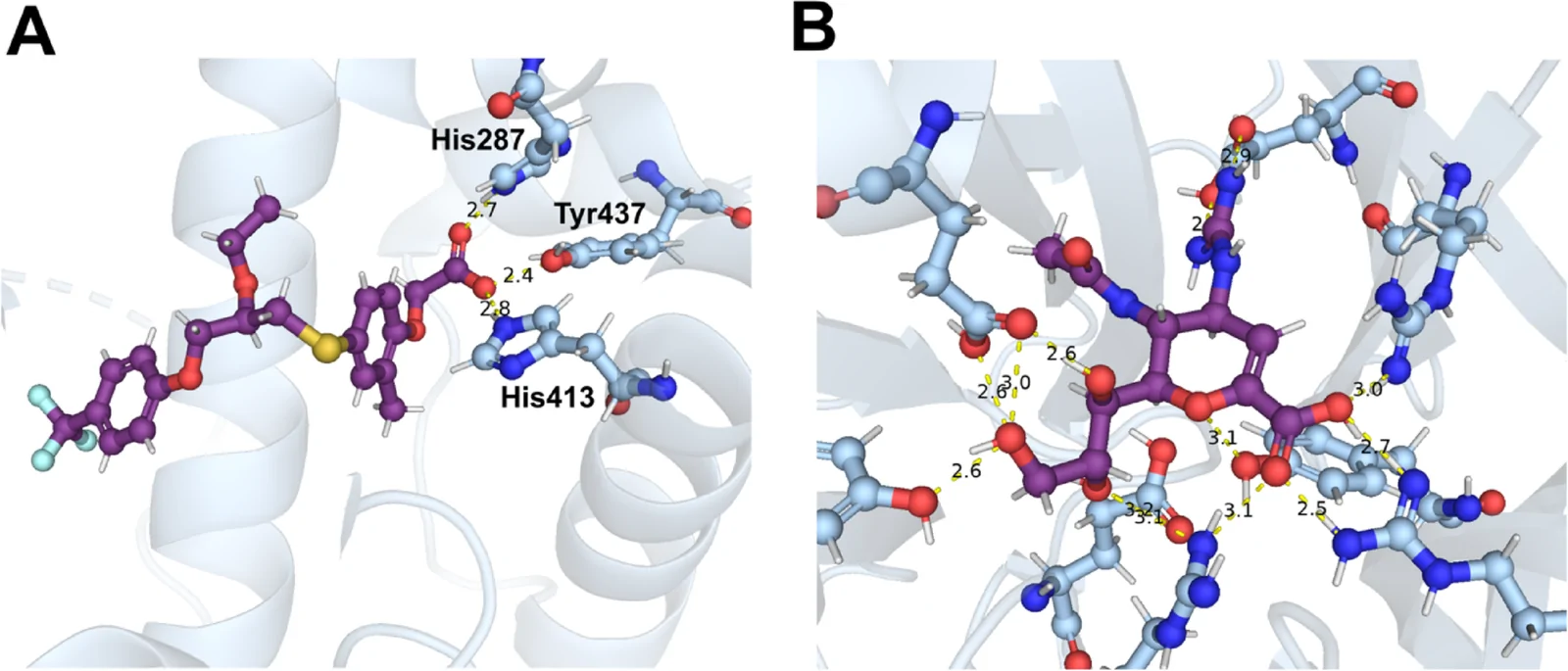
Hydrogen bond interactions between ligands and control proteins observed in experimental structures. **A** Seladelpar binding by Peroxisome proliferator-activated receptor delta (PDB: 8huo). **B** Zanamivir binding by Sialidase-2 (PDB: 2f0z). Ligand carbon atoms are shown in purple, while secondary structure elements and carbon atoms of interacting residues are shown in light blue. H-bonds appear as yellow dotted lines, with numbers indicating distances in Å

To assess the stability of the trajectories we calculated the following parameters: protein backbone Root Mean Square Deviation (RMSD), protein-ligand Center of Mass (COM) distance, number of protein-ligand hydrogen bonds, number of protein-ligand contacts and ligand Solvent-Accessible Surface Area (SASA). For each metric we derived ten statistical features from the time series (Materials and Methods), giving 50 features per trajectory. Most trajectories from T-group 188.1.1 showed protein backbone RMSD values below 2-2.5 Å (Supplementary Fig. 2), including the PPARα and PPARγ complexes (means 1.2-1.7 Å). Significant exceptions are 1ie9 (Vitamin D3 receptor) and 3hvl (Nuclear receptor subfamily 1 group I member 2) with maximum RMSD 3.5 Å and 2.8 Å respectively. Most trajectories from T-group 5.1.3 showed protein backbone RMSD values below 2 Å (Supplementary Fig. 3). The outliers are 1ijq (Low-density lipoprotein receptor) and 4gnb (Regucalcin) with maximum RMSD 3.5 Å and 2.7 Å respectively. Protein-ligand COM distance fluctuations for all trajectories from nuclear receptor T-group (188.1.1) were stable and did not exceed 2 Å (Supplementary Fig. 4), with one exception: in one of the two PPARγ (8hup) replicates the ligand moved abruptly at approximately 4 ns, the COM distance increasing from ~ 8 to ~ 13 Å and remaining displaced for the remainder of the simulation, accompanied by a loss of hydrogen bonds. The second PPARγ replicate remained stable throughout, despite both replicates being initiated from comparably ranked AlphaFold3 models. In T-group 5.1.3, TRIM2 (7qrv) and TRIM3 (7qrw) fluctuated by more than 3 Å (Supplementary Fig. 5), indicating unstable ligand placement [37]. Hydrogen-bond counts between Seladelpar and the nuclear-receptor complexes reached at most seven (Supplementary Fig. 6). The on-target control (8huo) did not form the most. Several complexes, including 1pq9, 1pzl and 1fcy, sustained higher mean counts, and the two PPARα (8hun) models formed the most of all (mean 4.0-4.8) despite PPARα being only weakly activated by Seladelpar. The two PPARγ (8hup) models formed few (mean 1.1-1.9). PyMOL detected at least three H-bonds between Seladelpar and residues of the experimental structure of Peroxisome proliferator-activated receptor delta (Fig. 2A). In contrast, the maximum number of hydrogen bonds between Zanamivir and 6-bladed beta-propeller ECOD T-group (5.1.3) was 14 and it was observed only for control protein Sialidase-2 (Supplementary Fig. 7). The number of H-bonds defined by PyMOL in experimental structure of Sialidase-2 with Zanamivir was 13 (Fig. 2B). Trajectories of all other proteins from this group showed significantly smaller number of H-bonds during the simulation (Supplementary Fig. 7).

For the nuclear-receptor group (188.1.1), the number of protein-ligand contacts (ligand atoms within 4 Å of the protein) stayed close to the total number of ligand atoms for every complex throughout the simulation (Supplementary Fig. 8), so this metric was nearly constant across the dataset and did not distinguish the complexes. In the second group, the contact count was highest for the control (mean 45), with Myocilin (4wxq) and Regucalcin (4gnb) at means of 35 and 40, respectively (Supplementary Fig. 9). The Solvent Accessible Surface Area (SASA) of the ligands revealed stable dynamics during the entire simulation period for both protein groups (Supplementary Figs. 10 and 11).

**Table 3:** Distance of each Seladelpar complex to the on-target control in the standardized 50-feature space

Receptor	PDB	Model	Group	Distance to nearest control
FXR	1osh	1	Tested negative	3.8
RARγ	1fcy	1	Tested negative	5
FXR	1osh	2	Tested negative	5.21
ERRγ	2e2r	2	Tested negative	5.58
RARγ	1fcy	2	Tested negative	5.87
ERRγ	2e2r	1	Tested negative	6.22
PPARα	8hun	2	Weak positive	6.23
PPARα	8hun	1	Weak positive	7.17
PPARγ	8hup	1	Weak positive	7.26
LXRβ	1pq9	1	Untested	7.77
LXRβ	1pq9	2	Untested	8.08
HNF4α	1pzl	1	Untested	8.44
PXR	3hvl	2	Untested	8.62
RXRα	1xvp	2	Untested	8.92
HNF4α	1pzl	2	Untested	9.23
RXRα	1xvp	1	Untested	9.81
VDR	1ie9	1	Untested	10.39
VDR	1ie9	2	Untested	11.97
PXR	3hvl	1	Untested	13.67
PPARγ	8hup	2	Weak positive	16.36

Euclidean distance from each complex to the nearer of the two control (PPARδ, 8huo) replicates, computed after standardizing all 50 trajectory-derived features; values are dimensionless. Models 1 and 2 are the two independent AlphaFold3 models simulated for each complex. Group indicates experimental outcome: “tested negative”, complexes tested with no detectable functional activity; “weak positive”, the PPARα and PPARγ comparators, which are weakly activated by Seladelpar; “untested”, candidates for which no experimental data are available. For reference, the two control replicates are separated by 4.58 in the same space

**Fig. 3:**
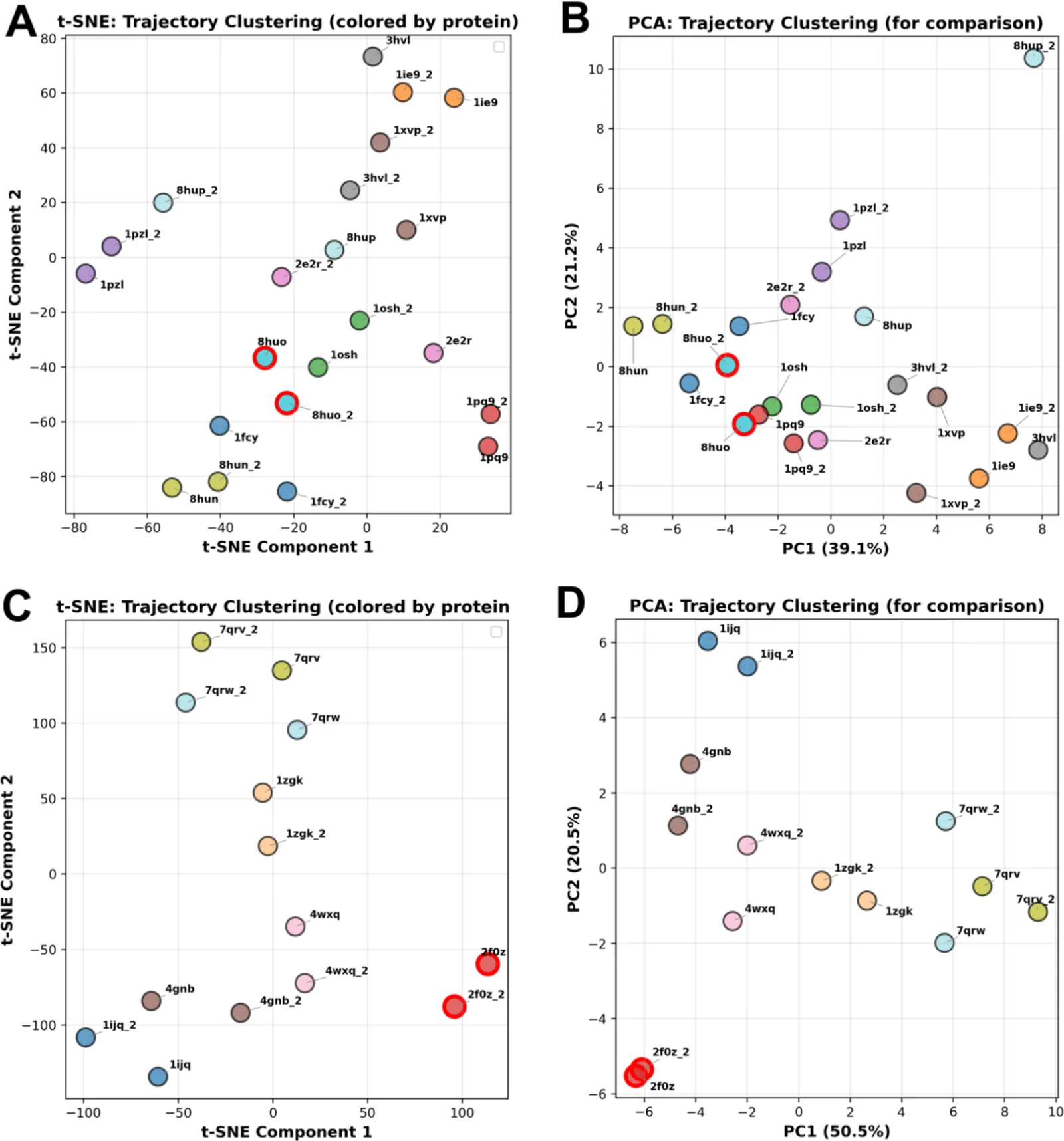
Clustering analysis of protein-ligand complexes based on MD-derived statistical features. **A** t-SNE and **B** PCA plot for protein-ligand complexes with Seladelpar. **C** t-SNE and **D** PCA plot for protein-ligand complexes with Zanamivir. Each circle represents a MD simulation trajectory. Circles outlined in red represent control proteins in each group. Labels above each protein-ligand complex indicate the PDB identifier, and, where present, a “_2” suffix denoting the second MD run performed on an independent AlphaFold3 model

Overall, we obtained 50 features per MD trajectory (5 metrics x 10 statistical features). PCA and t-SNE were used to visualize obtained features for both protein groups (Fig. 3A-D). Proximity of points on t-SNE and PCA plots indicates similarity across the 50 statistical features describing conformational dynamics during MD simulations. Because t-SNE preserves only local structure and its distances are not quantitative, we additionally computed Euclidean distances to the control in the standardized 50-feature space (Table 3; the full standardized feature matrix is provided in Supplementary Table 3). For the nuclear receptor T-group (188.1.1), the protein-ligand complexes with the most similar features to the control are: Bile acid receptor (PDB: 1osh), Retinoic Acid Receptor gamma (PDB: 1fcy), Estrogen-related receptor gamma (PDB: 2e2r) (Fig. 3A, B), the three subsequently selected for experimental testing. Two features of this ranking bound how finely it can be interpreted. First, the two control replicates were themselves separated by 4.58 units in the standardized space, exceeding the distance from the control to the closest-ranked complex (1osh, 3.80). For four of the eleven receptors, the separation between the two independent AlphaFold3 models exceeded the complex’s distance to the control (Supplementary Table 1), and three of these four are among the closest-ranked complexes, so the members placed nearest the control are also those least reproducible between replicates. Second, the weak-positive comparators PPARα and PPARγ, which are weakly activated by Seladelpar, ranked further from the control than several of the other candidates (Table 3), rather than closer as their known activity might suggest. The ranking therefore does not resolve differences smaller than the variation between replicate models, and its relationship to experimental activity is examined in Sect.  3.3. We further asked whether any single descriptor distinguished the control from the complexes that were subsequently tested and found inactive (Supplementary Table 2, Sect.  3.3). Of the 50 features, seven had non-overlapping ranges between the two groups, but five derive from the protein-ligand contact count, which stays close to the ligand atom count (52) in every trajectory and varies by only one to three atoms. The two remaining, minimum ligand SASA and minimum COM distance, differ by margins comparable to the variation between replicates. A further six features were invariant across all trajectories. No individual descriptor separated the control cleanly, and the feature set spans substantially fewer than 50 independent dimensions, with 55 of the 1,225 feature pairs correlated at |r| > 0.9.

For the 6-bladed beta-propeller T-group (5.1.3), the control protein is located further from other members than in the previous group, suggesting lower overall similarity in ligand binding dynamics or less conserved binding site behavior. The most similar trajectories in this case are: Myocilin (PDB: 4wxq), Regucalcin (PDB: 4gnb), Kelch-like ECH-associated protein 1 (PDB: 1zgk) (Fig. 3C, D). As no experimental testing was performed for this group, these rankings are presented as a computational extension of the workflow to a second, unrelated fold rather than as validated predictions.

### Experimental validation of predicted Seladelpar off targets

**Fig. 4:**
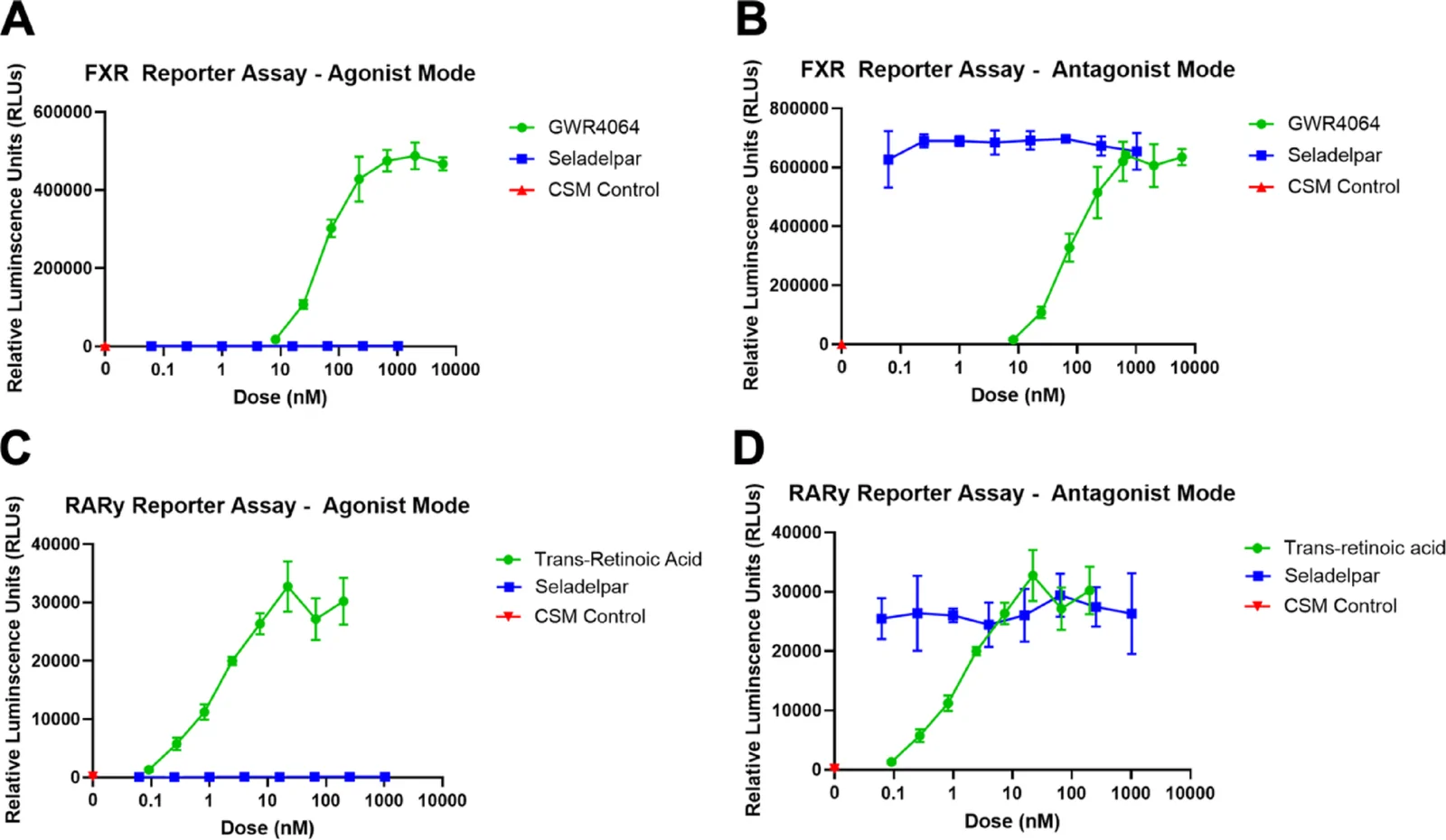
Reporter assay validation of predicted Seladelpar off targets in the nuclear receptor T-group. Across all panels, Seladelpar is shown in blue, the reference agonist in green, the negative control (compound screening media alone; CSM) in red. **A** FXR reporter assay in agonist mode, with GW4064 as the reference agonist. **B** FXR reporter assay in antagonist mode; Seladelpar was added to wells containing GW4064 at its EC_80_ dose. **C** RARγ reporter assay in agonist mode, with trans-retinoic acid as the reference agonist. **D** RARγ reporter assay in antagonist mode; Seladelpar was added to wells containing trans-retinoic acid at its EC_50_ dose

**Fig. 5:**
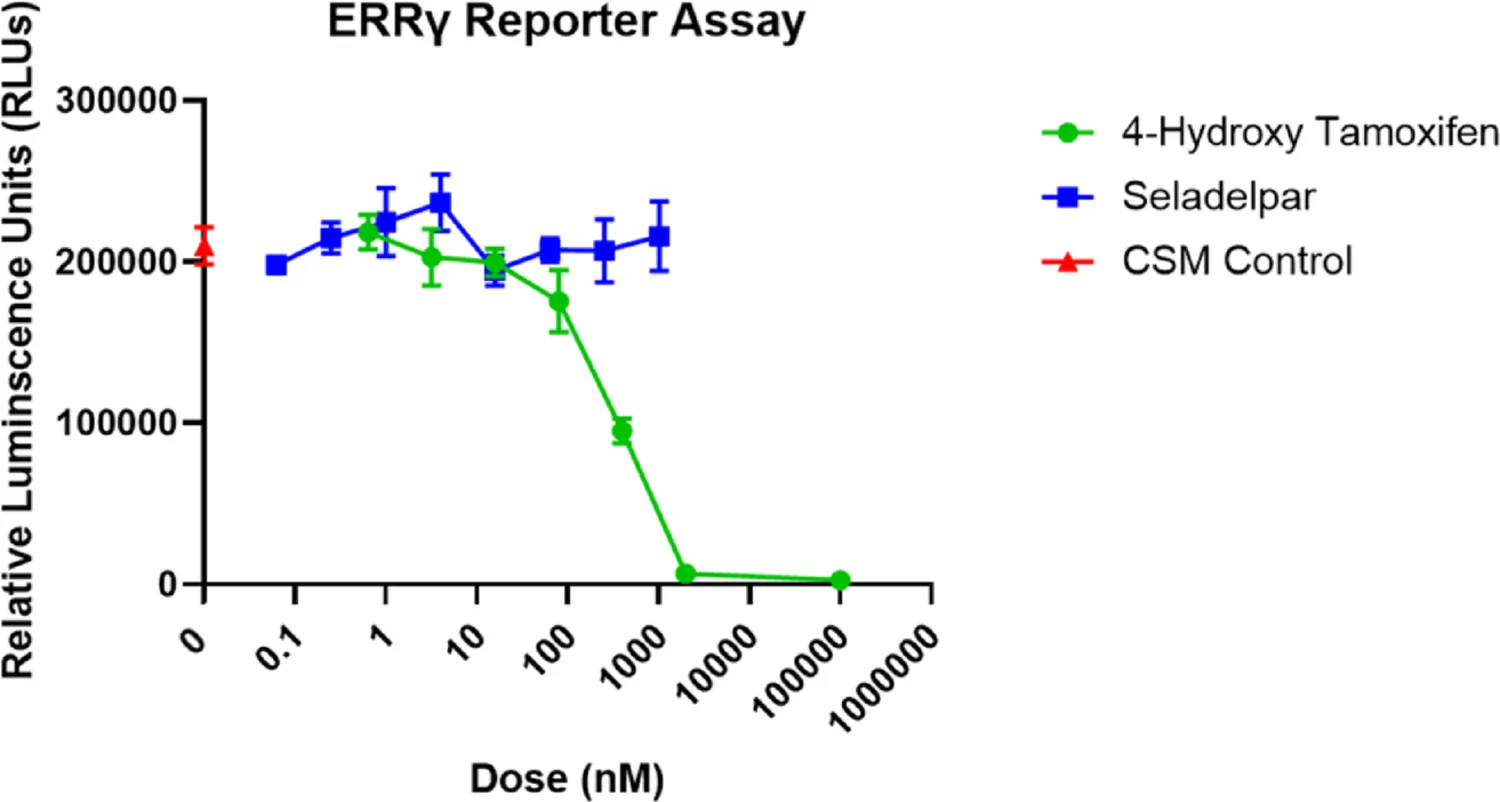
ERRγ reporter assay in agonist and inverse-agonist mode. Seladelpar is shown in blue, the reference inverse agonist 4-hydroxy tamoxifen in green, and the CSM (compound screening medium) negative control in red

To test whether trajectory-similarity clustering predicts off-target interactions, we evaluated the three top-ranked candidates from the nuclear receptor T-group using two complementary approaches: cell-based luciferase reporter assays to assess functional activity, and thermal shift assays to assess direct ligand binding to purified protein. The three candidates identified by clustering analysis were Bile acid receptor (FXR; PDB: 1osh), Retinoic acid receptor gamma (RARγ; PDB: 1fcy), and Estrogen-related receptor gamma (ERRγ; PDB: 2e2r). Reporter assays were performed using commercial engineered mammalian reporter cell lines (Indigo Biosciences, State College, PA, USA). FXR and RARγ were evaluated in both agonist and antagonist modes (Fig. 4), whereas ERRγ activity was assessed exclusively in agonist/inverse-agonist mode due to the constitutive expression of active ERRγ with the reporter cell line (Fig. 5). Antagonist mode was used to detect potential displacement of a reference agonist at its EC80/EC50 (FXR: GW4064; RARγ: trans-retinoic acid).

Despite the predicted similarity in MD-derived trajectory features, Seladelpar did not exhibit functional activity against any of the three nuclear receptors (Figs. 4 and 5). Agonist mode signals for Seladelpar did not rise above the negative control across the full tested concentration range, indicating that Seladelpar does not activate FXR, RARγ, or ERRγ. In antagonist mode, Seladelpar failed to inhibit GW4064-mediated FXR activation or trans-retinoic acid-mediated RARγ activation at their respective EC80/EC50 doses, indicating that Seladelpar does not displace the reference agonist at either receptor. Similarly, ERRγ did not exhibit inverse-agonist activity (Fig. 5).

**Fig. 6:**
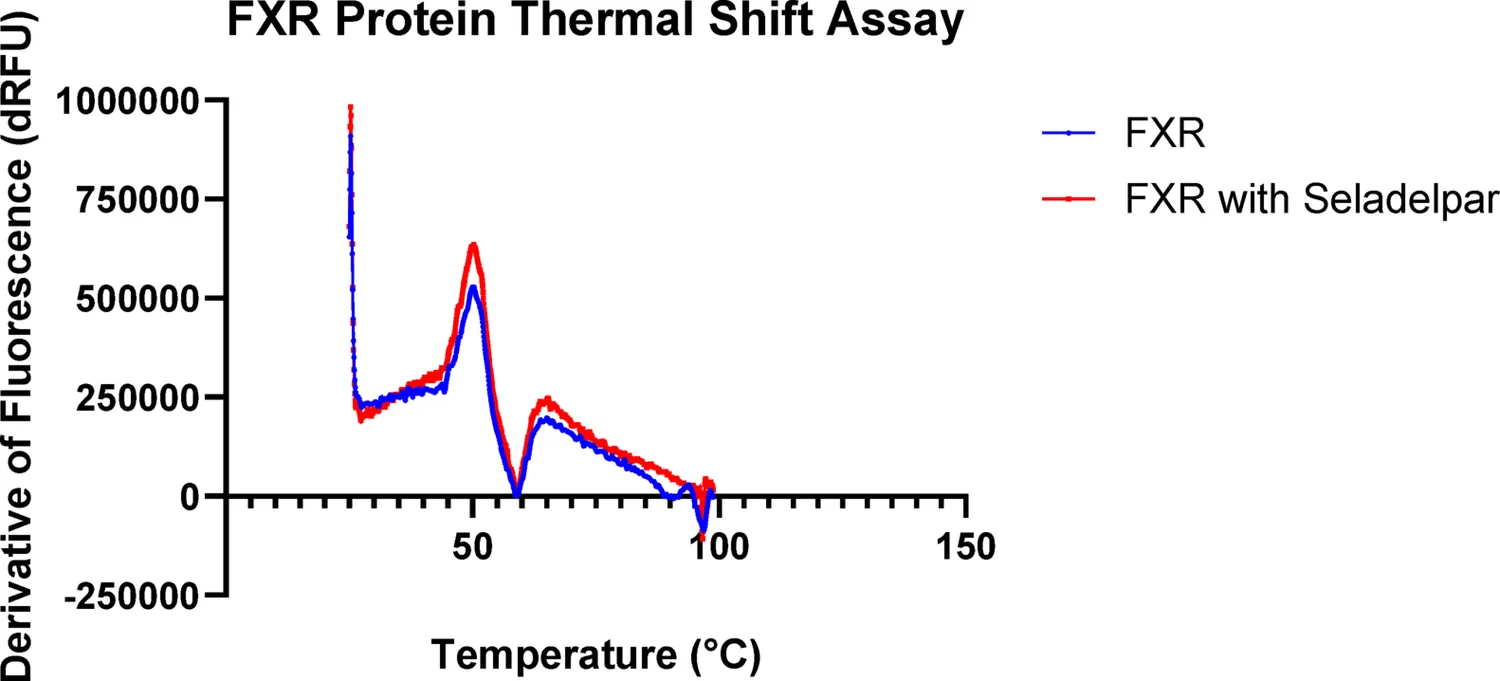
Thermal shift assay of FXR with and without Seladelpar. Derivative plot (dRFU vs. temperature) of FXR melt curves under optimized conditions (1 µg protein per well, 1× dye). FXR alone is shown in blue and FXR with Seladelpar in red

To further assess whether Seladelpar physically binds FXR or RARγ, we performed thermal shift assays with purified recombinant protein. Melting temperatures (Tm) were extracted from derivative plots (dRFU vs. temperature) using a nonlinear fit with a Boltzmann sigmoidal regression model. Initial experiments revealed elevated baseline fluorescence and required optimization of dye concentration and protein loading. Under the optimized conditions (1 µg protein per well, 1× dye) FXR alone produced a reproducible melting transition with a Tm of approximately 55 °C (Supplementary Fig. 12). Addition of Seladelpar yielded a Tm of 54.8 °C compared to 54.5 °C for FXR alone (Fig. 6), corresponding to a ΔTm of 0.3 °C. Combined with the absence of any functional response in the reporter assays, the thermal shift data provide no evidence of Seladelpar binding to FXR. For RARγ, no usable melting transition was obtained under any of the conditions tested, including the dye titration series (Supplementary Fig. 13). We were therefore unable to assess Seladelpar binding to RARγ by this method. Thermal shift validation was not performed for ERRγ. Taken together, these assays show that Seladelpar does not functionally activate or inhibit these three receptors under the conditions tested. For FXR, the thermal shift assay additionally provides no indication of direct binding. Direct binding measurements were not obtained for RARγ or ERRγ, so weak or non-functional binding to these two receptors cannot be excluded on the basis of the present data.

### AlphaFold3-predicted binding modes diverge from canonical agonist binding

**Fig. 7:**
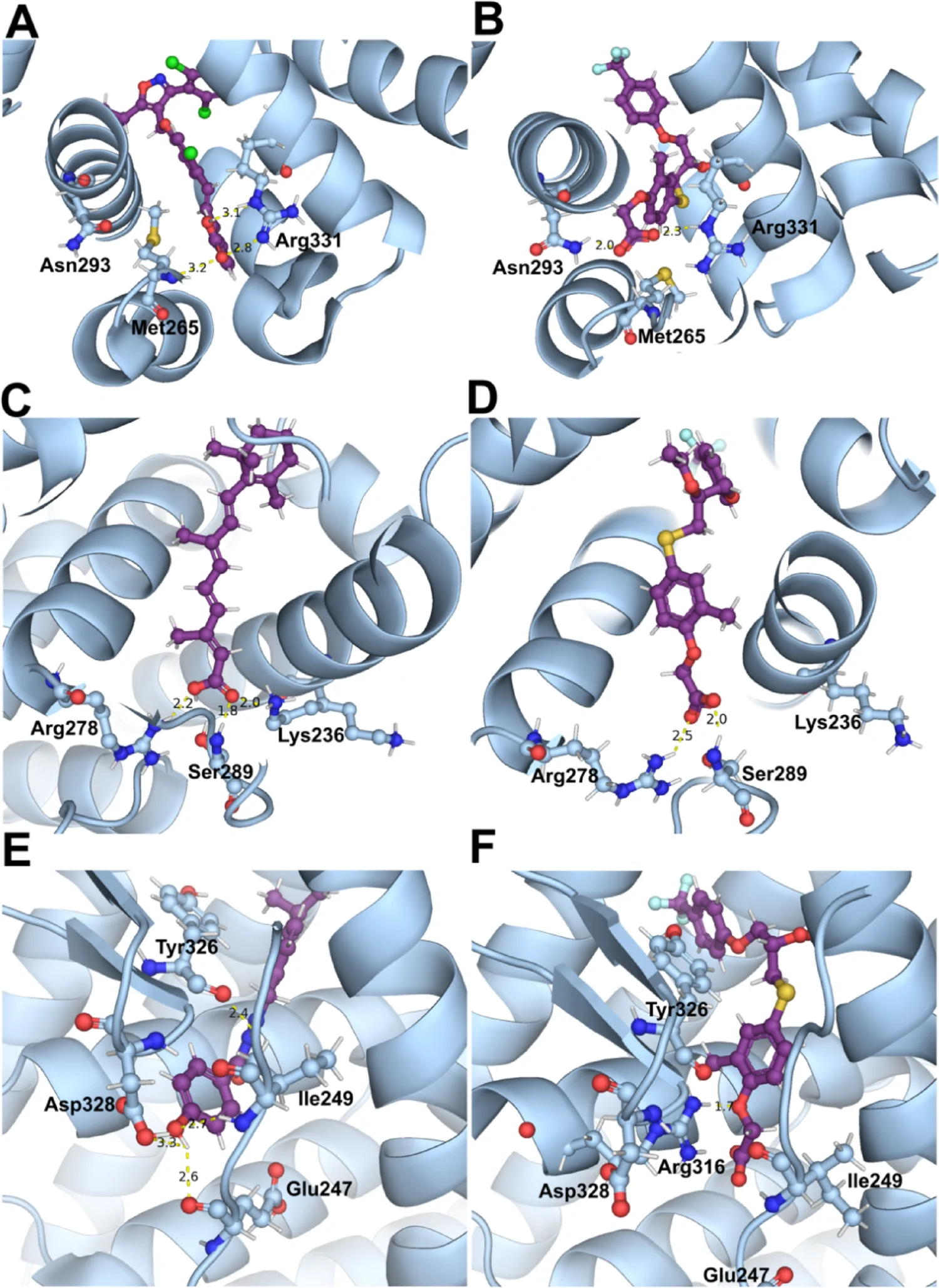
Comparison of experimental agonist binding modes with AlphaFold3 predicted Seladelpar binding modes for three predicted off-target receptors. Ligand carbon atoms are colored purple; interacting residues are shown in light blue with oxygen (red), nitrogen (blue), and sulfur (yellow) colored by element. Hydrogen bonds were identified using PyMOL polar contact detection and are shown as yellow dashed lines with distances in Å. **A** FXR with GW4064 (PDB: 3DCT). **B** AlphaFold3 model of FXR with Seladelpar. **C** RARγ with retinoic acid (PDB: 2LBD). **D** AlphaFold3 model of RARγ with Seladelpar. **E** ERRγ with GSK4716 (PDB: 2GPP). **F** AlphaFold3 model of ERRγ with Seladelpar

The experimental verification using reporter and thermal shift assays revealed that FXR, RARγ, and ERRγ do not show detectable functional engagement with Seladelpar, despite stable MD trajectories and global metrics close to the on-target PPARδ control, which is what placed them near the control in the clustering (Fig. 3A, B). Total hydrogen bond counts gave no warning either and some of the off-target candidates formed more H-bonds with Seladelpar than the control did (Supplementary Fig. 6). To identify a structural basis for the absence of functional activity, we compared how Seladelpar is positioned in each off-target model with the corresponding experimental structure of the same receptor bound to a known agonist (Fig. 7).

In the experimental FXR structure with GW4064 (PDB: 3DCT; Fig. 7A), the agonist’s carboxylate forms two hydrogen bonds between both NH groups of Arg331 and different oxygens of the carboxylate (3.1 and 2.8 Å), with an additional contact to Met265 (3.2 Å). This two-point Arg interaction is a signature of FXR agonist binding [38]. In the AF3 model (Fig. 7B), the carboxylate of Seladelpar reaches Arg331, but only with a single hydrogen bond. Although Seladelpar forms additional polar contacts with Asn293, this residue does not engage GW4064 in the experimental structure. Thus, the specific interaction that defines FXR agonism is replaced by a different set of contacts that the receptor does not use in its binding with the known agonist GW4064.

The RARγ pocket is mostly hydrophobic, with a small polar cluster (Lys236, Ser289, and Arg278) at the carboxylate-binding end [39, 40]. In the X-ray crystal structure with retinoic acid (PDB: 2LBD [41]; Fig. 7C), the ligand carboxylate forms hydrogen bonds with all three residues. In the experimental RARγ-retinoic acid crystal structure (PDB: 2LBD), the Lys236 side chain is resolved in two alternate conformations, with the electron density consistent with the side chain occupying one position approximately 59% of the time and a second position 41% of the time. Only the second conformation places the ε-amino group within hydrogen-bonding distance of the ligand carboxylate (Fig. 7C). In the AF3 model of RARγ with Seladelpar (Fig. 7D), the drug carboxylate occupies a position similar to that of the retinoic acid carboxylate in 2LBD but with a slightly different rotational angle. The carboxylate forms hydrogen bonds with Arg278 and Ser289, reproducing two of the three canonical polar contacts, but the altered angle places the carboxylate out of reach of Lys236, leaving the polar cluster incomplete.

Unlike FXR and RARγ, the ERRγ binding pocket has been characterized primarily with phenol-containing ligands. The synthetic ERRγ agonist GSK4716 (PDB: 2GPP; Fig. 7E) [42] binds through its phenolic hydroxyl group, which is positioned within hydrogen-bonding distance of Tyr326, Glu247, Asp328, and the backbone amide of Ile249. In the AF3 model of ERRγ with Seladelpar (Fig. 7F), the drug’s carboxylate group occupies the pocket but does not engage these phenol-recognition residues. Whether a carboxylate group can engage ERRγ in a manner similar to a phenolic hydroxyl is not established in the published literature, but the AF3 model does not reproduce the canonical phenol-binding geometry.

**Fig. 8:**
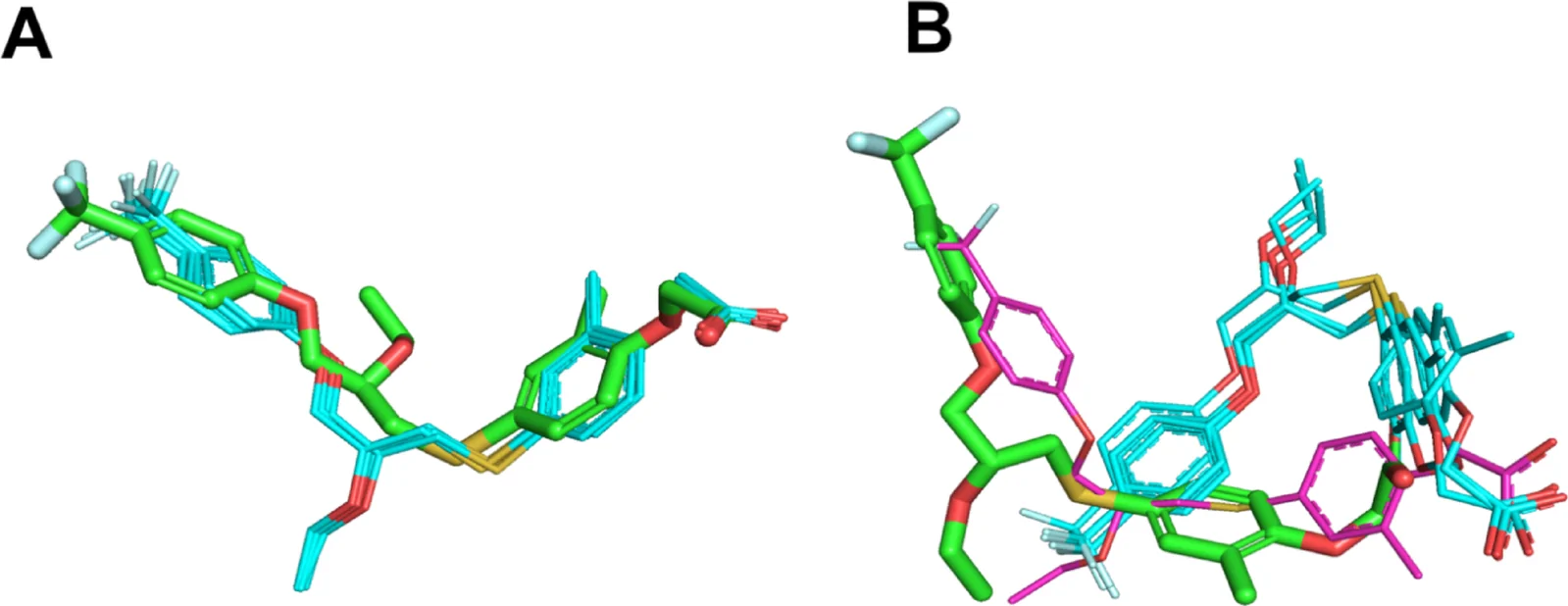
Superposition of AlphaFold3-predicted Seladelpar poses on the experimental drug-bound structures of PPARα and PPARγ. The five AlphaFold3 models of each complex (cyan) are shown superposed on the experimental Seladelpar-bound crystal structure (green) after alignment of the protein. **A** PPARα (experimental structure PDB: 8HUN): the five models overlay closely on the experimental pose. **B** PPARγ (experimental structure PDB: 8HUP): four models (cyan) adopt an orientation differing from the experimental pose, while the model with the lowest AlphaFold3 ranking score (magenta) lies closest to it

For two receptors, PPARα and PPARγ, an experimental structure of the Seladelpar complex is available (PDB: 8HUN and 8HUP [29]), allowing the predicted binding modes to be compared directly with the drug-bound crystal structure rather than with a different agonist. Both structures were deposited in December 2022, after the AlphaFold3 training cutoff, and were absent from the template database used here (PDB snapshot of 28 September 2022). The predicted poses therefore represent genuine predictions. For PPARα, all five AlphaFold3 models reproduced the experimental Seladelpar pose closely, with ligand RMSDs of 2.3-2.6 Å after protein superposition (Fig. 8A). For PPARγ, the models were both less accurate and less consistent: four of the five placed the ligand in an orientation differing markedly from the experimental pose (RMSD 7.9-8.3 Å), and only the fifth approached the correct binding mode (3.5 Å; Fig. 8B). This fifth model was the one AlphaFold3 ranked lowest by confidence (ranking score 0.71, versus 0.76-0.77 for the other four). For PPARγ, the four highest-ranked models converged on the incorrect pose, while the single accurate model was ranked lowest and would have been excluded by any confidence-based selection. This is only apparent in hindsight, because an experimental Seladelpar structure exists for PPARγ. For a genuine off-target prediction, where no such structure is available, model confidence is the only prospective signal, and here it favored the incorrect pose. Filtering models on confidence before simulation would therefore not have improved the predictions, and in this case would have removed the one accurate pose. The divergence in ligand placement is not explained by errors in the modelled receptor. Across all six receptors with an available experimental complex, the Cα RMSD over binding-site residues ranged from 0.37 to 2.84 Å (Supplementary Table 4), comparable to the whole-domain deviations. Within PPARγ, pocket accuracy did not predict pose accuracy: the model with the most accurate binding site (1.29 Å) placed the ligand 8.3 Å from its experimental position, while the single model reproducing the experimental binding mode (3.5 Å) had one of the least accurate pockets (1.67 Å).

To test whether this behavior was specific to AlphaFold3, we predicted both PPAR complexes with Boltz-2 [43]. For PPARα, Boltz-2 placed the ligand close to its experimental position (ligand RMSD 2.96-3.14 Å across five models); for PPARγ, none of its five models recovered the experimental binding mode (5.02-5.35 Å), although they converged more tightly than the AlphaFold3 models (Supplementary Table 5). Neither method reproduced the PPARγ pose, indicating that the difficulty is a property of the target rather than of a single prediction method.

## Conclusions

In this study we investigated whether ECOD-guided AF3 modeling combined with molecular dynamics trajectory analysis could identify functional off-targets for approved drugs. The approach was applied to two systems: Seladelpar with the nuclear receptor T-group (188.1.1) and Zanamivir with the 6-bladed β-propeller T-group (5.1.3). The Seladelpar predictions were taken forward into experimental validation, while the Zanamivir set was analyzed computationally only. Ranking the complexes by the similarity of their trajectory-derived features to the on-target PPARδ control placed FXR, RARγ and ERRγ closest to the control, and several candidates formed more hydrogen bonds with Seladelpar than the control did. None of the three showed measurable functional activity in reporter assays, and the one candidate for which a direct binding measurement was obtained (FXR) showed no detectable thermal stabilization by Seladelpar. Two things limit how much weight the ranking can carry. First, the two independent models of the control differed from each other more than the control differed from its closest-ranked candidate. Second, PPARα and PPARγ, which are known to be weakly activated by Seladelpar, ranked further from the control than the candidates that turned out to be inactive.

When we checked how Seladelpar was positioned in each off-target pocket compared to the experimental agonist binding mode, the same pattern emerged in each receptor, with different specifics. In FXR and RARγ AF3 models, the carboxylate group was located close to the canonical position used by GW4064 and retinoic acid, respectively. However, the exact geometry of hydrogen bonds was different: a single hydrogen bond to Arg331 instead of two in FXR, and a small rotational shift in RARγ that placed the carboxylate out of reach of Lys236. In the case of ERRγ, every published agonist-bound structure features a phenolic hydroxyl, whereas the AF3 model placed Seladelpar’s carboxylate within the ligand-binding pocket but outside the canonical phenol-recognition residues engaged by GSK4716.

Where experimental Seladelpar complexes were available, the predicted poses could be assessed directly. AlphaFold3 reproduced the PPARα pose (ligand RMSD 2.3-2.6 Å) but not the PPARγ pose, where four of five models were displaced by 7.9-8.3 Å and the single accurate model was the one ranked lowest by AlphaFold3’s own confidence score. Boltz-2 showed the same target-specific difficulty, recovering the PPARα pose but none of the PPARγ models. The modelled binding sites themselves were close to the experimental structures (Cα RMSD 0.37-2.84 Å), so the errors in ligand placement appeared despite the pocket being reproduced accurately.

In our two systems, the descriptors we used (backbone RMSD, center-of-mass distance, hydrogen-bond and contact counts, and ligand SASA) tracked the overall stability of each complex but did not separate binding modes that support receptor activation from those that do not. A stable trajectory, a low RMSD and a reasonable hydrogen-bond count tell us that the predicted complex holds together, not that it is functionally engaged. All three off-target candidates looked stable by these measures, yet none produced detectable functional activity, and the incomplete polar networks described above were not reflected in any of the descriptors This is a limitation of these particular descriptors rather than of MD-based approaches in general.

Several limitations of this work should be acknowledged. First, the thermal shift assay was only successful for FXR. We were unable to obtain a usable melting transition for RARγ under the conditions tested, and we did not perform thermal shift validation for ERRγ. The reporter assays are therefore the primary line of evidence for the absence of functional activity, with thermal shift providing direct binding confirmation only for FXR. Experimental validation covers three candidates from a single drug system, so these results do not establish the overall performance of the workflow. Second, the assays used here cannot formally exclude very weak binding below their detection limits, although the absence of any functional response in the cellular assays argues against such interactions being biologically meaningful. Third, each predicted pose was simulated in a single 30 ns trajectory. Extending the PPARα and PPARγ complexes to 100 ns did not change the trajectory-derived features, but a single trajectory per pose limits what can be concluded about stability.

## Supplementary Information

Below is the link to the electronic supplementary material.

Supplementary Material 1

Supplementary Material 2

Supplementary Material 3

Supplementary Material 4

Supplementary Material 5

Supplementary Material 6

## Data Availability

The PDB structures used in this study are available from the RCSB Protein Data Bank (https://www.rcsb.org). ECOD domain classifications are available from the ECOD database (http://prodata.swmed.edu/ecod/). The AlphaFold3 model coordinates, MD simulation input files (topologies and parameter files), and analysis scripts generated in this study are available from the corresponding author upon reasonable request. Raw experimental data from reporter assays and thermal shift assays are available from the corresponding author upon reasonable request. Molecular dynamics trajectory files are not made publicly available due to their large size but can be regenerated from the deposited input structures using the GROMACS protocols described in the Methods.
