# Supplementary material for: Stable simulations do not guarantee functional engagement: a case study of off-target prediction for Seladelpar and Zanamivir
Source: J Comput Aided Mol Des. 2026 Sep 3;40(1):227. doi: 10.1007/s10822-026-00936-w (PMC13541830; doi:10.1007/s10822-026-00936-w)
Supplement: Supplementary file 1 [file 10822_2026_936_MOESM6_ESM.pdf]

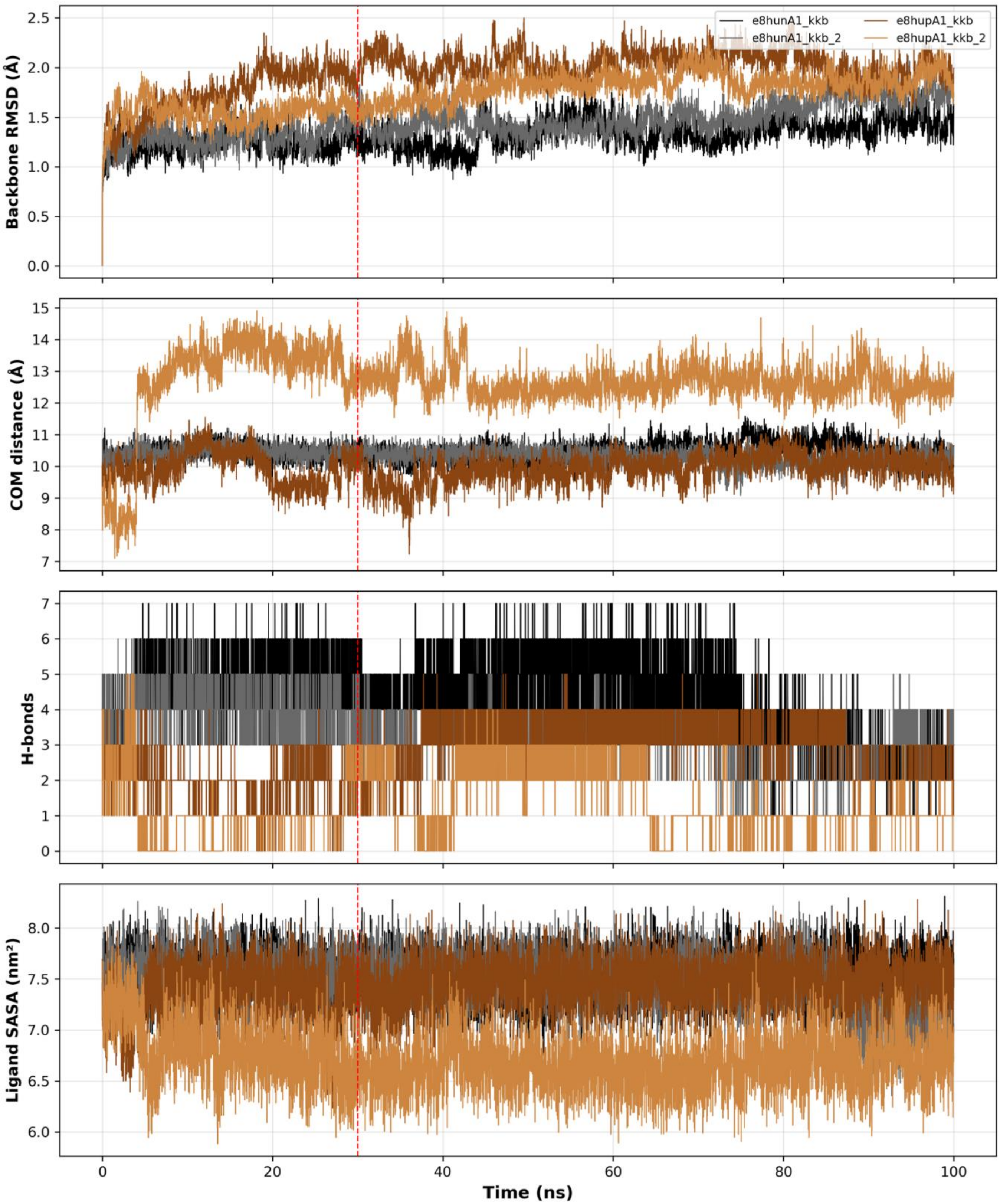

Supplementary Figure 1. Convergence of trajectory-derived metrics over 100 ns for the PPARα (8hun) and PPARγ (8hup) complexes. Backbone RMSD, protein–ligand centre-of-mass (COM) distance, hydrogen-bond count, and ligand solvent-accessible surface area (SASA) are shown for both

independent AlphaFold3 replicates of each complex (black and grey, PPARα; dark and light brown, PPARγ). The near-constant contact count, saturated at the ligand atom count, is omitted for clarity. The dashed red line marks 30 ns, the window used for the clustering analysis. Metric values over the first 30 ns are representative of the full trajectory.

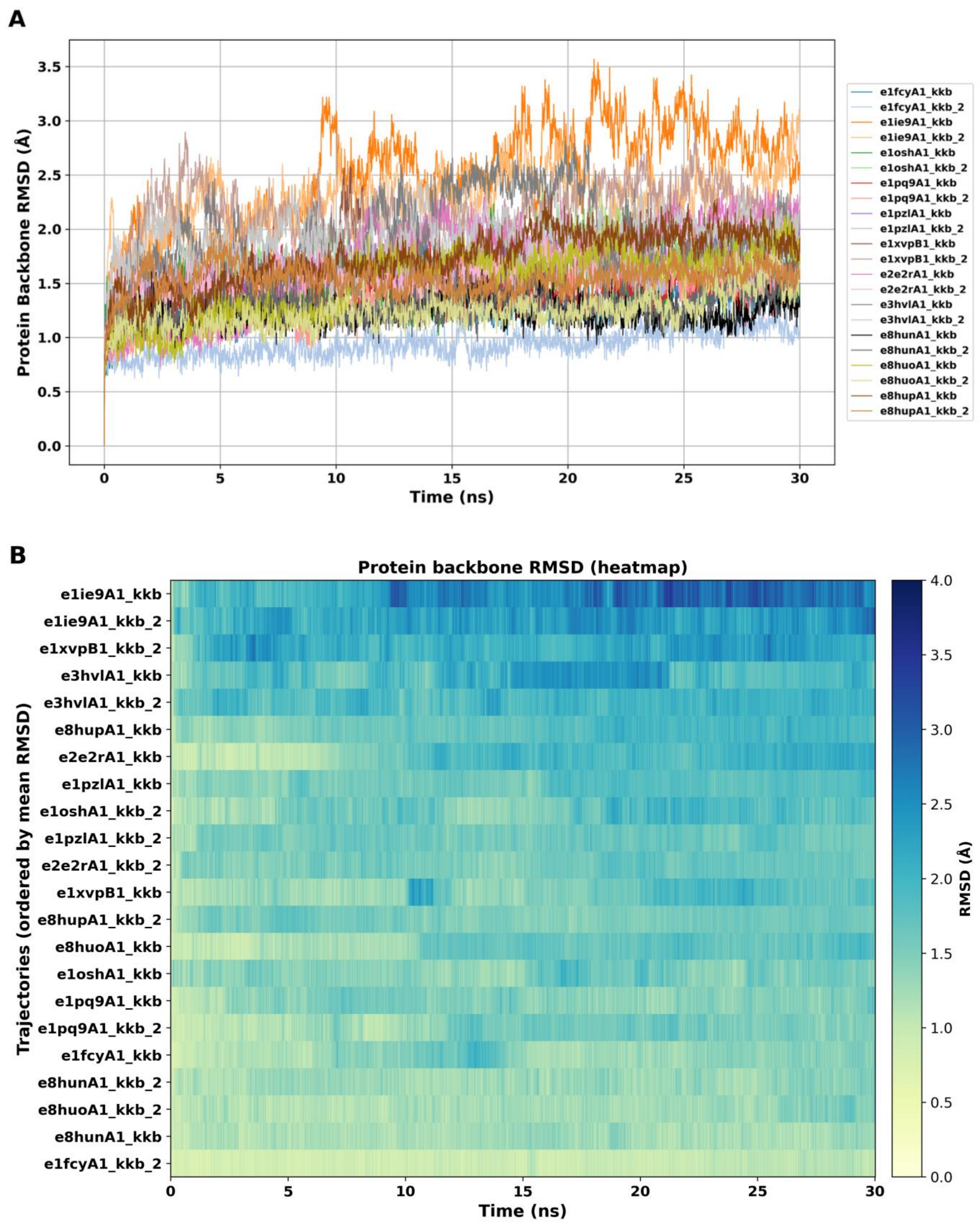

Supplementary Figure 2. Protein RMSD for proteins from ECOD T-group id 188.1.1 (Nuclear receptor ligand-binding domain) across all trajectories. (A) Linear graph representation. **(B)** Heatmap representation.

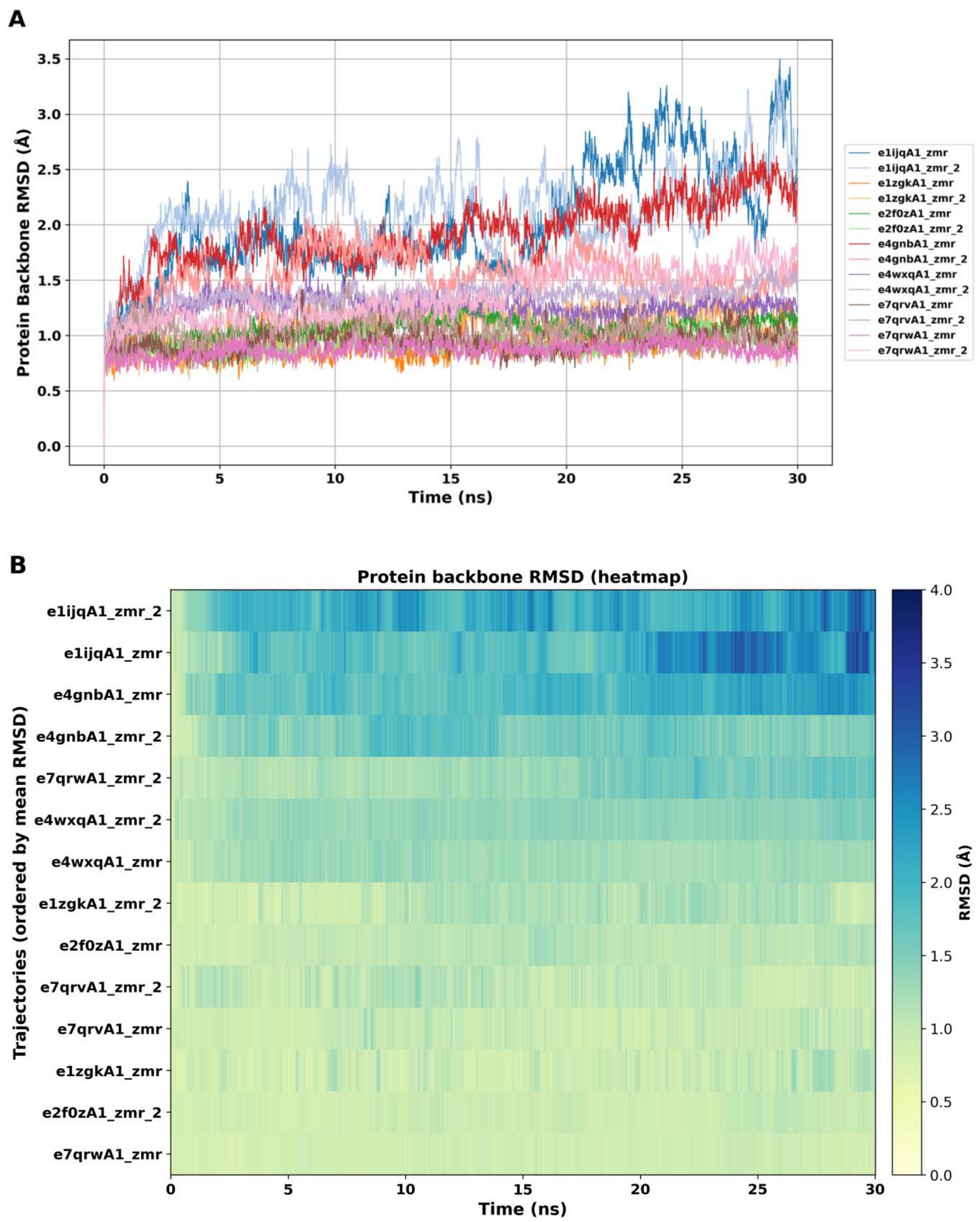

Supplementary Figure 3. Protein RMSD for proteins from ECOD T-group id 5.1.3 (6-bladed beta-propeller) across all trajectories. (A) Linear graph representation. **(B)** Heatmap representation.

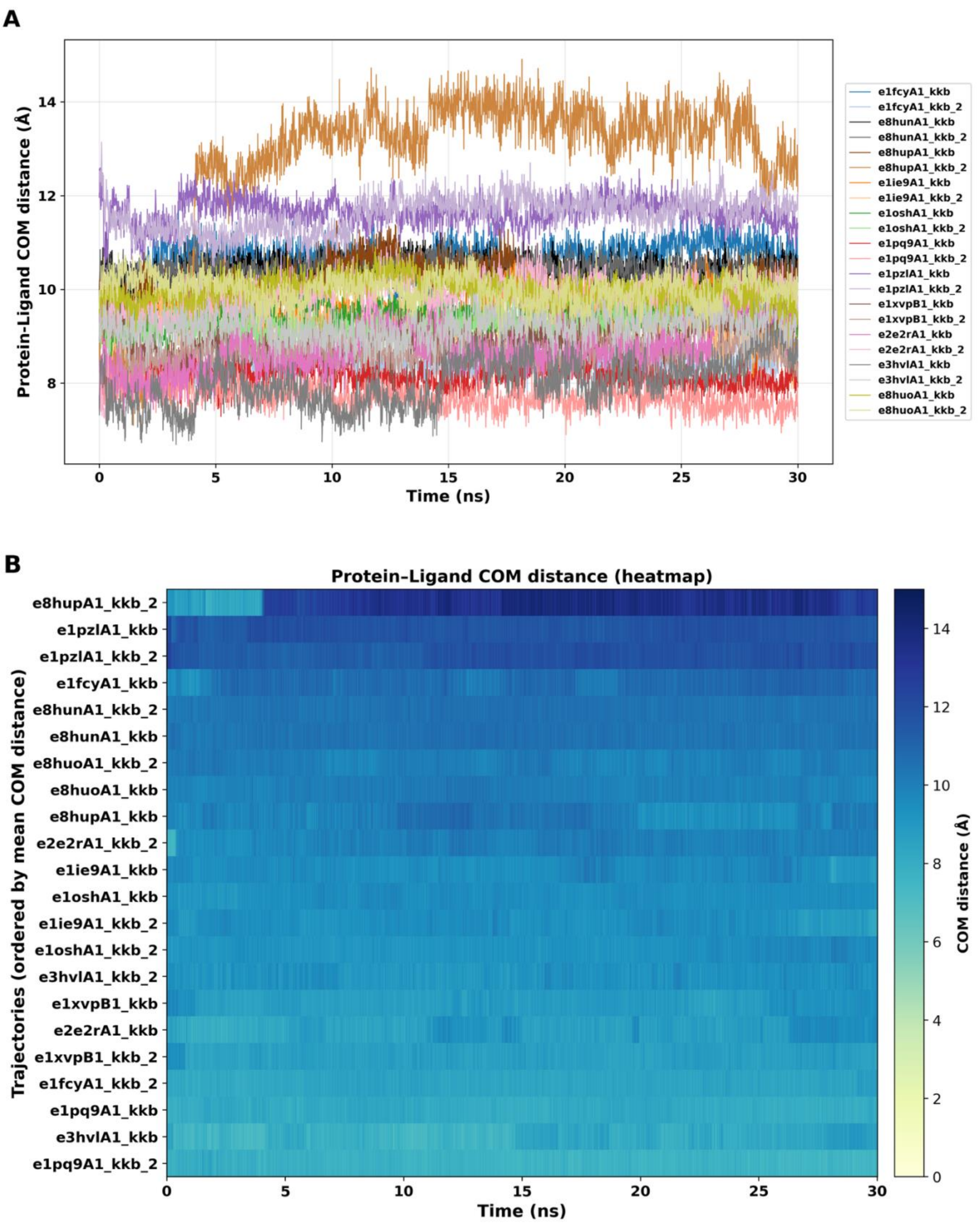

Supplementary Figure 4. Protein-ligand center of mass (COM) distance for proteins from ECOD T-group id 188.1.1 (Nuclear receptor ligand-binding domain) across all trajectories. (A) Linear graph representation. **(B)** Heatmap representation.

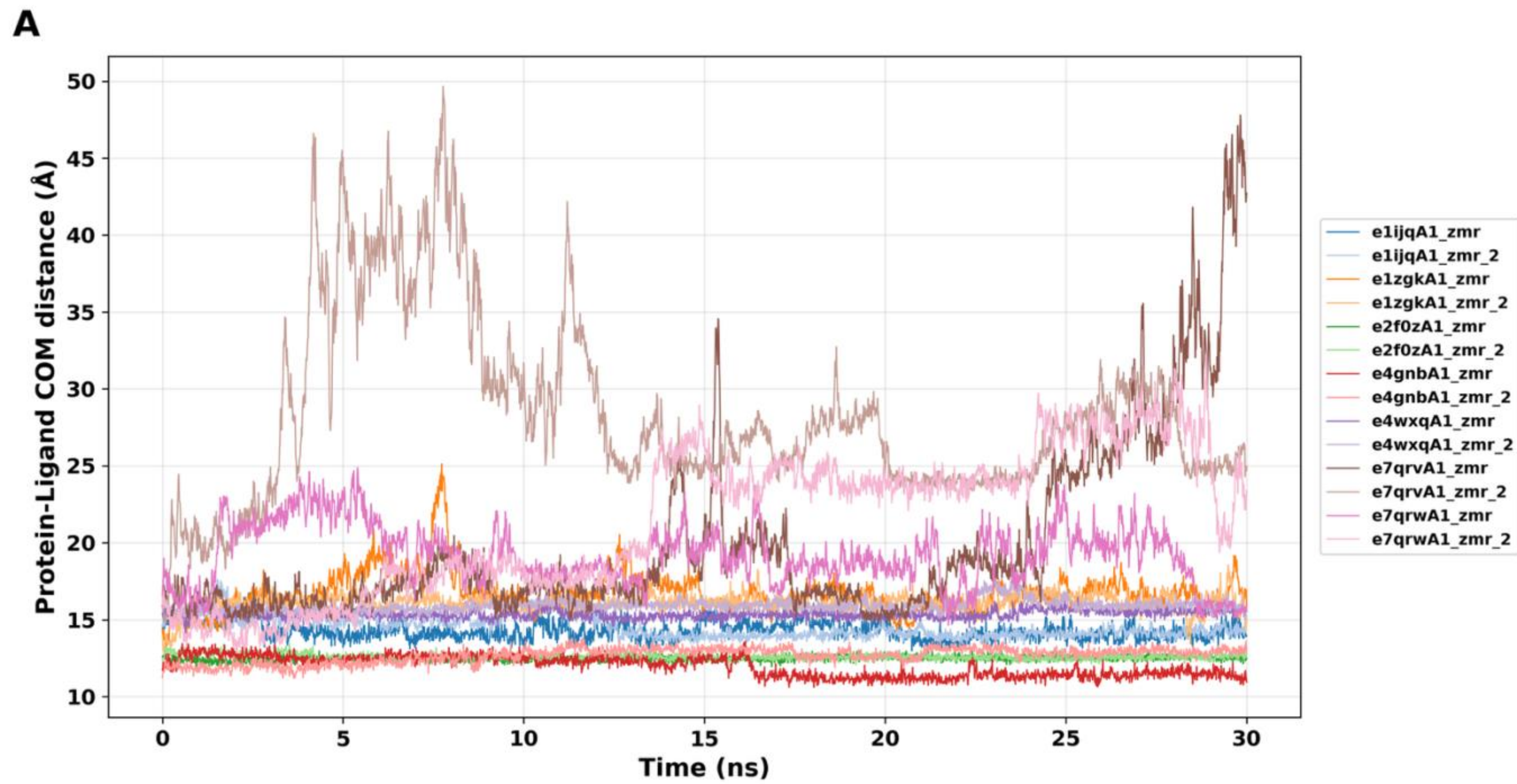

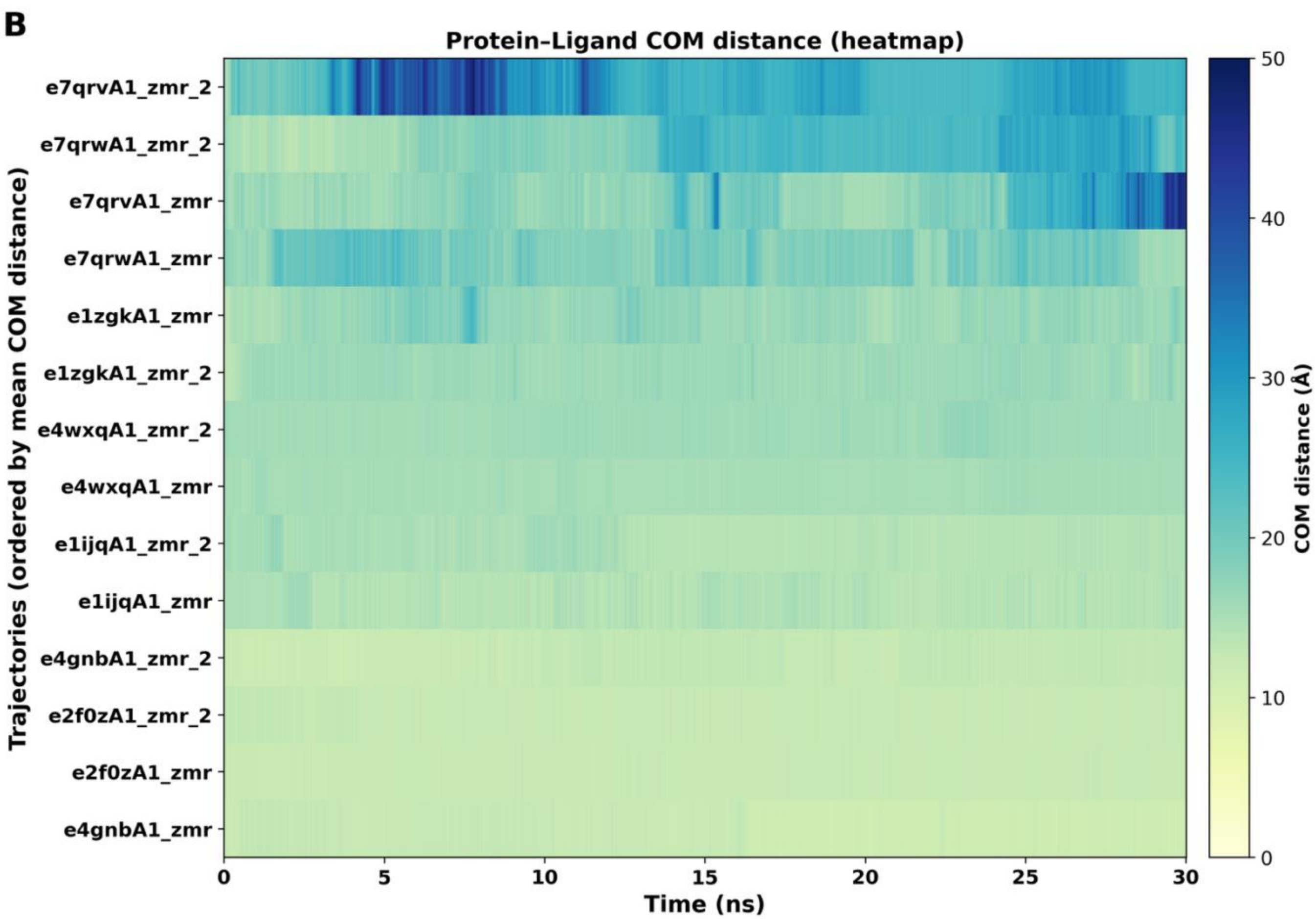

Supplementary Figure 5. Protein-ligand center of mass (COM) distance for proteins from ECOD T-group id 5.1.3 (6-bladed beta-propeller) across all trajectories. (A) Linear graph representation. **(B)** Heatmap representation.

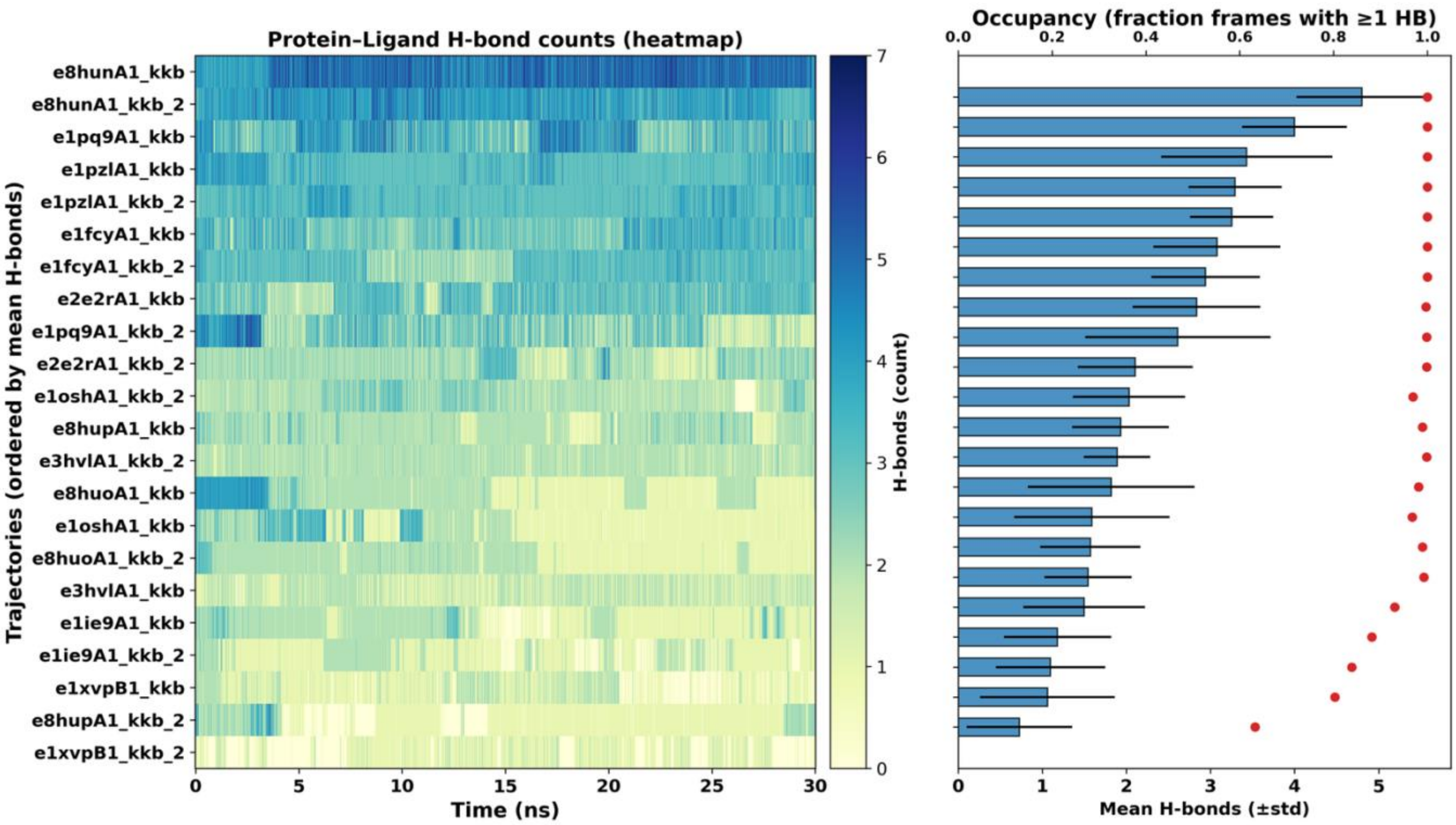

Supplementary Figure 6. Hydrogen bond dynamics between ligand (Seladelpar) and proteins from Nuclear receptor ligand-binding domain ECOD T-group. (Left) Heatmap of H-bond counts over time for all trajectories, ordered by mean H-bond count (highest at top). (Right) Mean H-bonds ± std (blue bars) and occupancy fraction (red circles, proportion of frames with ≥1 H-bond) for each trajectory.

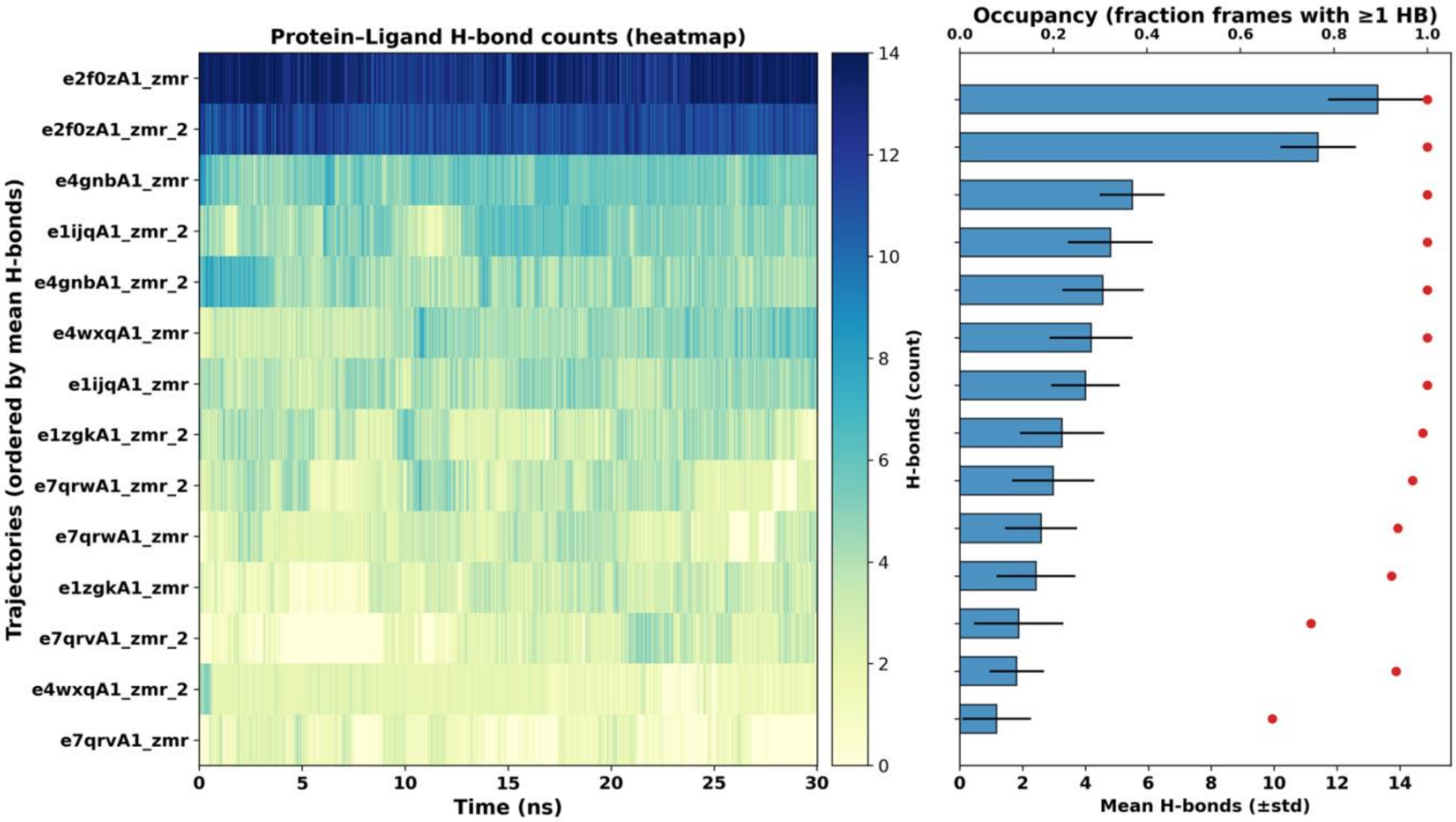

Supplementary Figure 7. Hydrogen bond dynamics between ligand (Zanamivir) and proteins from 6-bladed beta-propeller ECOD T-group. (Left) Heatmap of H-bond counts over time for all trajectories, ordered by mean H-bond count (highest at top). (Right) Mean H-bonds ± std (blue bars) and occupancy fraction (red circles, proportion of frames with ≥1 H-bond) for each trajectory.

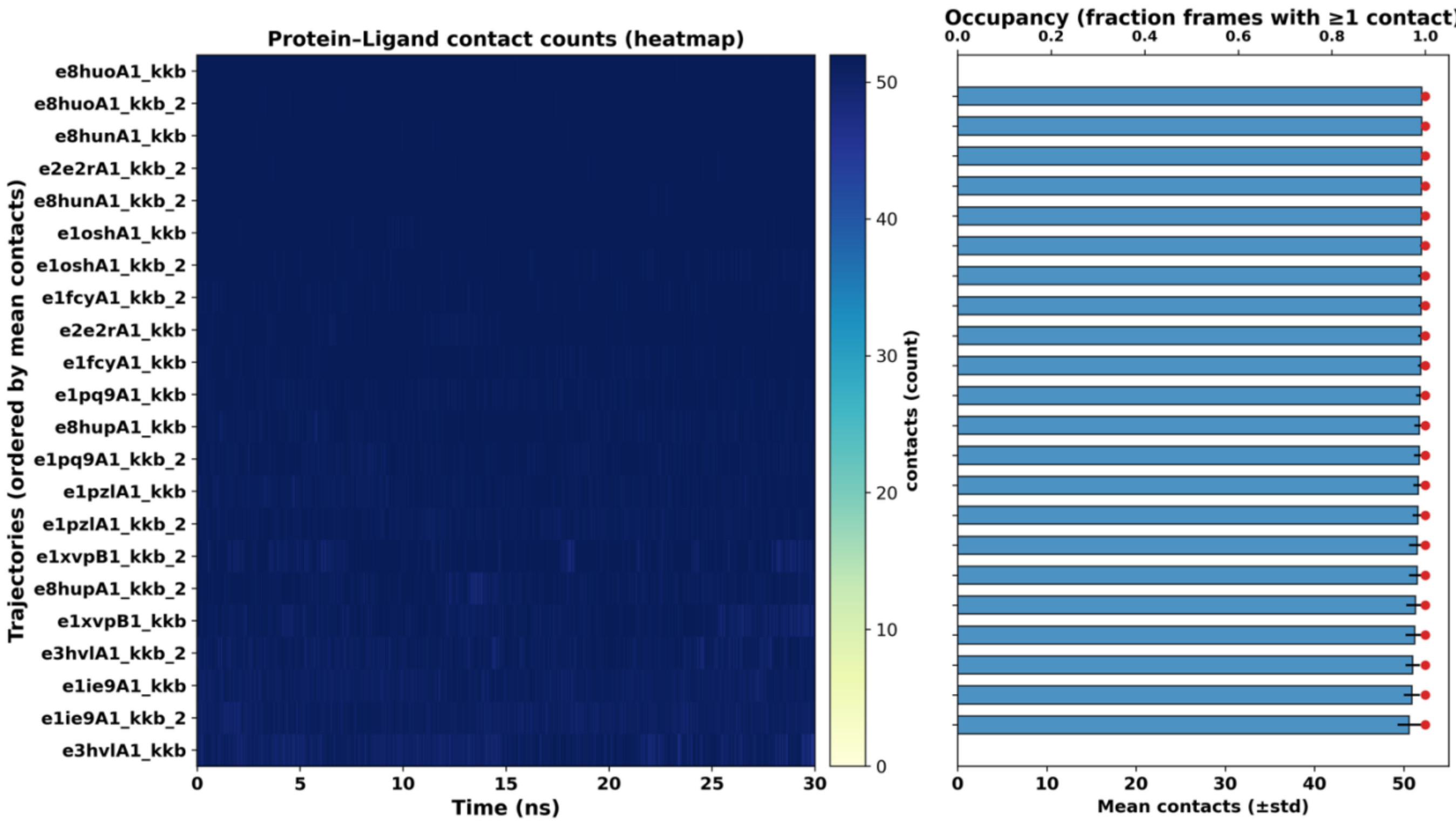

Supplementary Figure 8. Contact (≤ 4 Å) dynamics between ligand (Seladelpar) and proteins from Nuclear receptor ligand-binding domain ECOD T-group. (Left) Heatmap of contact counts over time for all trajectories, ordered by mean contact count (highest at top). (Right) Mean contacts ± std (blue bars) and occupancy fraction (red circles, proportion of frames with ≥1 contact) for each trajectory.

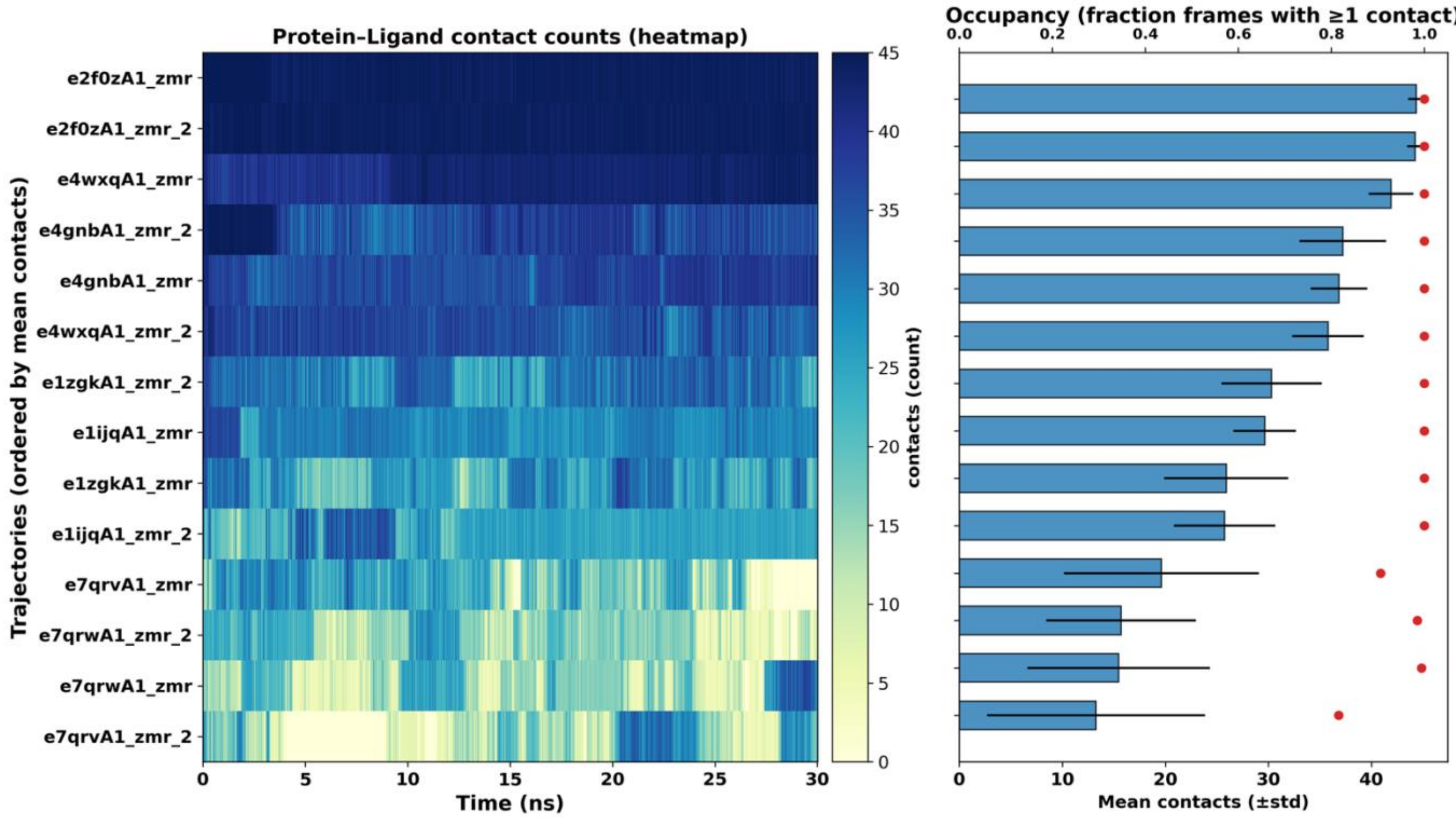

Supplementary Figure 9. Contact (≤ 4 Å) dynamics between ligand (Zanamivir) and proteins from 6-bladed beta-propeller ECOD T-group. (Left) Heatmap of contact counts over time for all trajectories, ordered by mean contact count (highest at top). (Right) Mean contacts ± std (blue bars) and occupancy fraction (red circles, proportion of frames with ≥1 contact) for each trajectory.

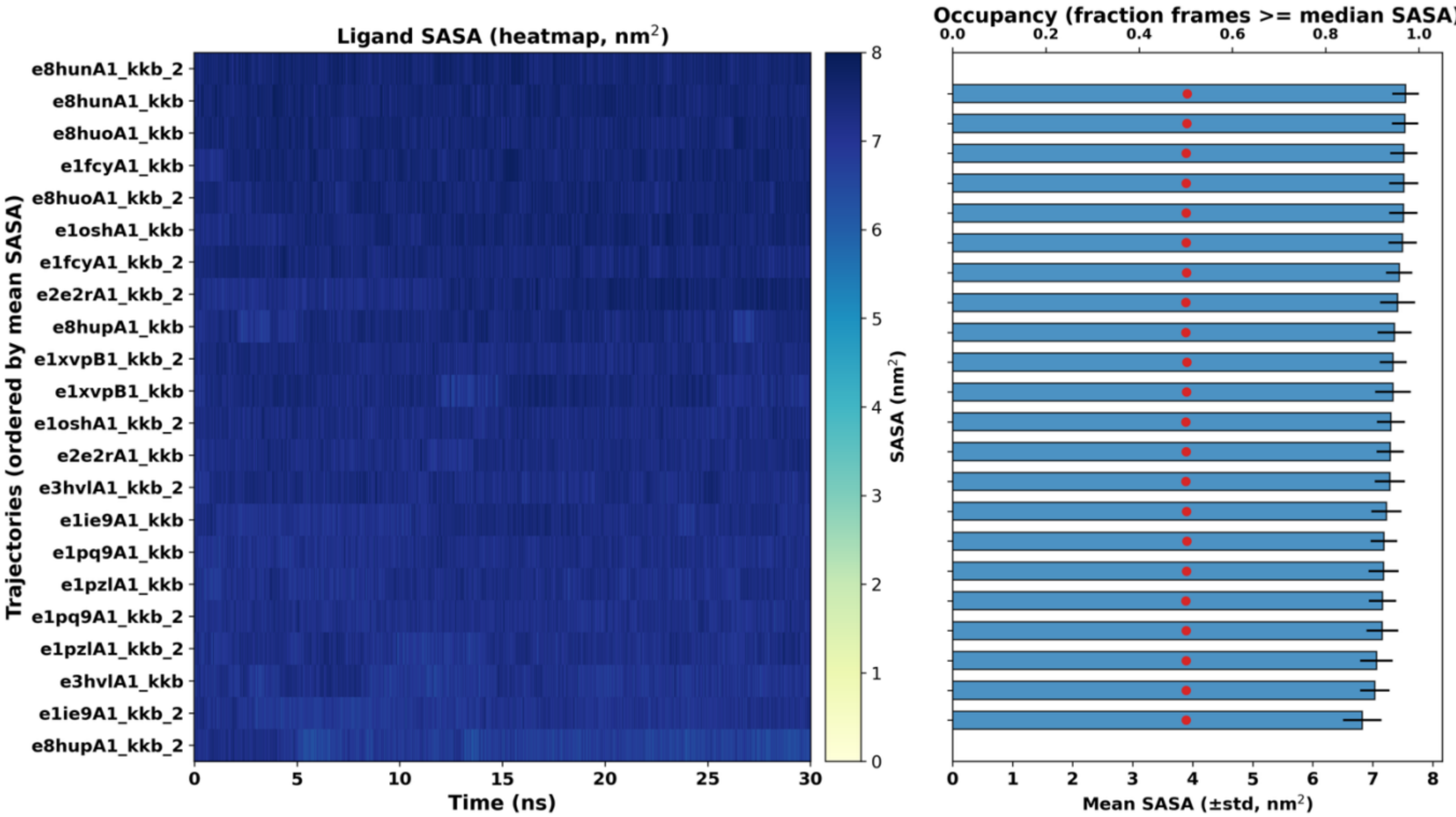

Supplementary Figure 10. Ligand Solvent Accessible Surface Area (SASA) dynamics for Seladelpar binding to Nuclear receptor ligand-binding domain proteins. (Left) Heatmap of SASA over time for all trajectories, ordered by mean SASA (highest at top). (Right) Mean SASA ± std (blue bars) and occupancy fraction (red circles, proportion of frames with SASA ≥ median SASA).

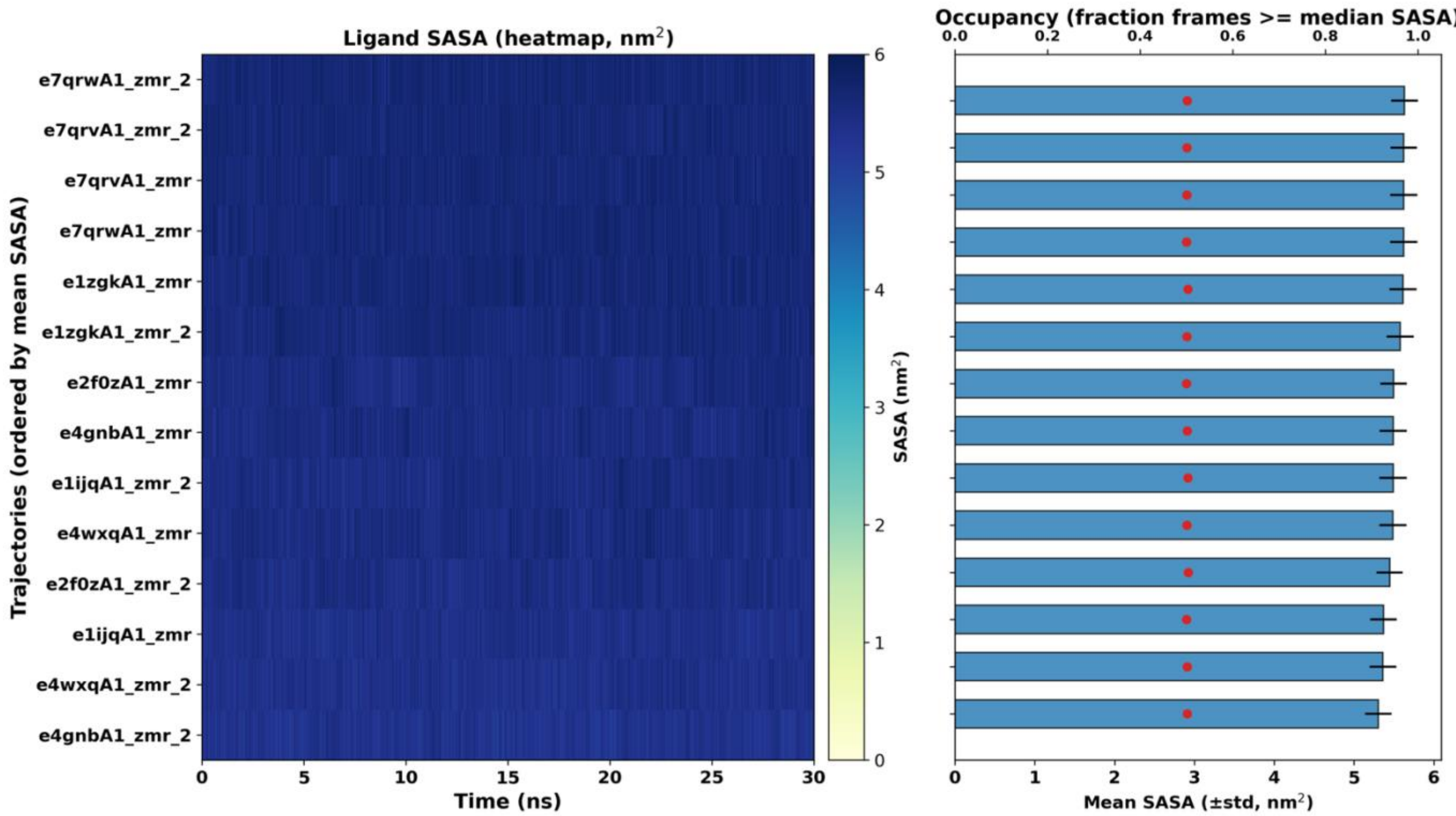

Supplementary Figure 11. Ligand Solvent Accessible Surface Area (SASA) dynamics for Zanamivir binding to proteins from 6-bladed beta-propeller ECOD T-group. (Left) Heatmap of SASA over time for all trajectories, ordered by mean SASA (highest at top). (Right) Mean SASA ± std (blue bars) and occupancy fraction (red circles, proportion of frames with SASA ≥ median SASA).

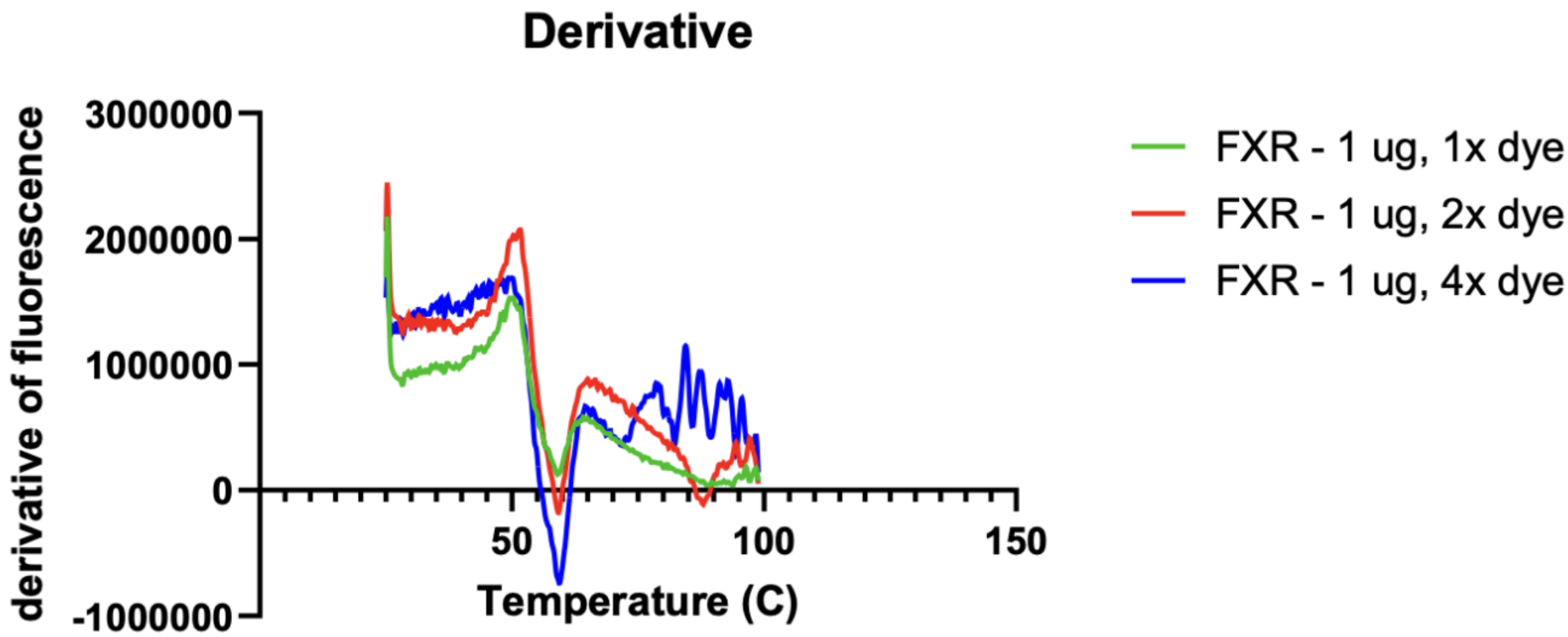

Supplementary Figure 12. Optimization of thermal shift assay conditions for FXR. Derivative plots (dF/dT vs temperature) of FXR melt curves across a dye titration series. FXR (1 µg per well) was assayed at 1× (green), 2× (red), and 4× (blue) dye concentration.

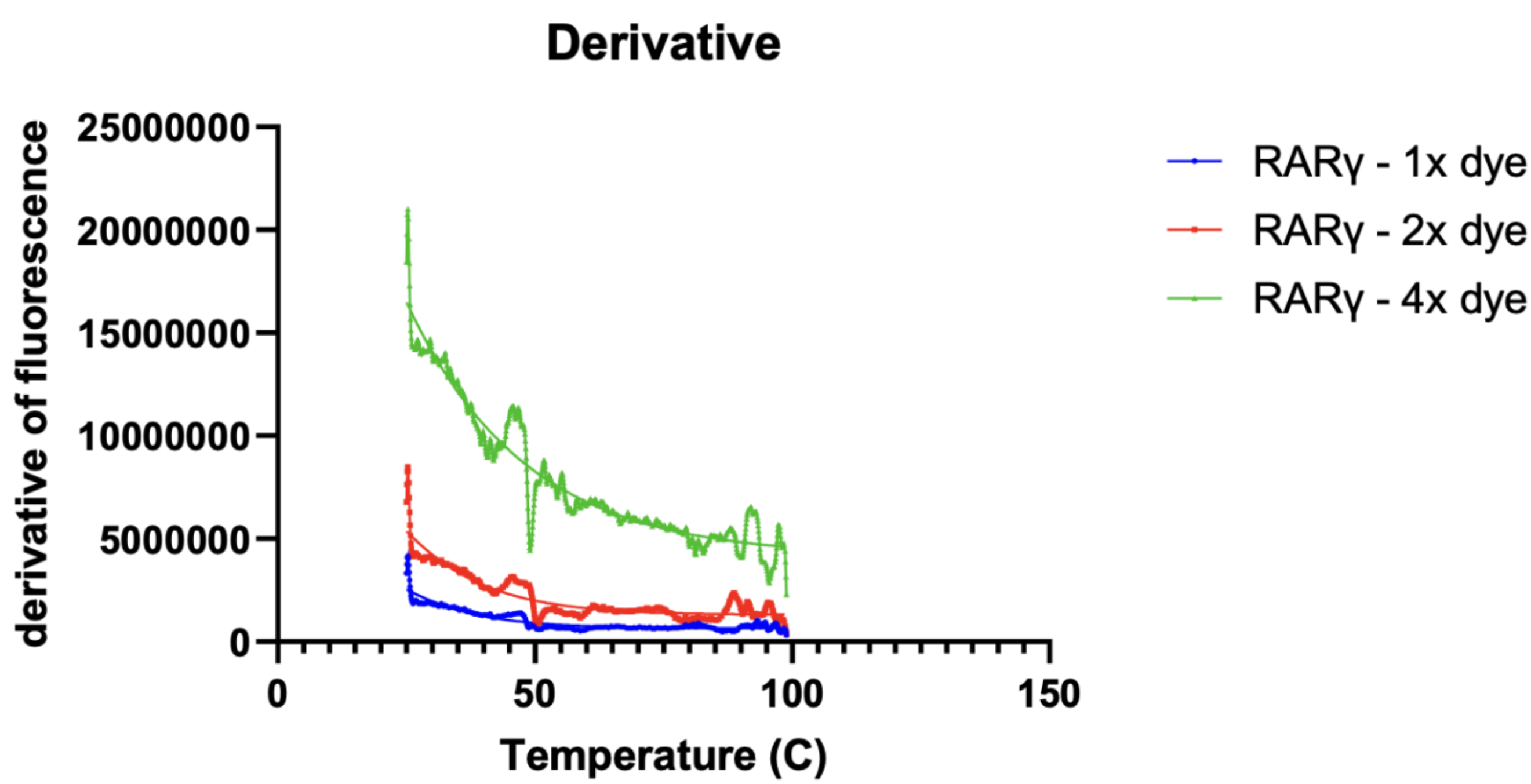

Supplementary Figure 13. RARγ thermal shift assay. Derivative plots (dF/dT vs temperature) of RARγ melt curves across a dye titration series. RARγ (1 µg per well) was assayed at 1× (blue), 2× (red), and 4× (green) dye concentration.
